# SCA: recovering single-cell heterogeneity through information-based dimensionality reduction

**DOI:** 10.1186/s13059-023-02998-7

**Published:** 2023-08-25

**Authors:** Benjamin DeMeo, Bonnie Berger

**Affiliations:** 1 Computer Science and Artificial Intelligence Laboratory, MIT, Cambridge, 02139 MA USA; 2 Department of Biomedical Informatics, Harvard University, Cambridge, 02138 MA USA; 3 Department of Mathematics, MIT, Cambridge, 02139 MA USA

## Abstract

Dimensionality reduction summarizes the complex transcriptomic landscape of single-cell datasets for downstream analyses. Current approaches favor large cellular populations defined by many genes, at the expense of smaller and more subtly defined populations. Here, we present surprisal component analysis (SCA), a technique that newly leverages the information-theoretic notion of surprisal for dimensionality reduction to promote more meaningful signal extraction. For example, SCA uncovers clinically important cytotoxic T-cell subpopulations that are indistinguishable using existing pipelines. We also demonstrate that SCA substantially improves downstream imputation. SCA’s efficient information-theoretic paradigm has broad applications to the study of complex biological tissues in health and disease.

**Supplementary Information:**

The online version contains supplementary material available at 10.1186/s13059-023-02998-7.

## Background

Single-cell RNA sequencing (scRNA-seq) produces transcript counts for individual cells, enabling fine-grained analyses of biological tissues. Single-cell datasets can uncover cellular populations and gene-gene interactions that play critical roles in biological and pathological phenomena [1–3]. Identifying and characterizing this heterogeneity is a key motivator of many single-cell experiments.

However, the size, high dimensionality, and noisiness of single-cell data complicates this task. Modern experiments profile tens of thousands of genes per cell, often with high dropout levels (under-sampling of mRNA molecules) and technical noise. Dimensionality reduction, whereby the data is represented in a lower-dimensional space with enriched signal, has become a cornerstone of modern scRNA-seq analysis pipelines. For example, principal component analysis (PCA) projects the data to a lower-dimensional linear subspace such that the total variance of the projected data is maximized. Independent component analysis (ICA) instead aims to identify non-Gaussian combinations of features. Both have found widespread use in single-cell studies [4–7]. A more recent method, scVI [8], models transcript counts using a zero-inflated negative-binomial distribution, and performs variational inference to non-linearly embed each cell into a low-dimensional parameter space. Graph-based dimensionality reduction methods such as PHATE [9] and diffusion maps [10] compute pairwise similarities between cells using diffusion on a *k*-nearest-neighbor network and create embeddings that preserve these similarities.

While these approaches are effective, they often fail to capture the full cellular diversity of complex tissues for two reasons. First, rare cell types, by definition, account for a small fraction of the observations, and therefore contribute little to a dataset’s global structure. Second, many distinctions between cellular populations hinge on just a few of the thousands of genes measured; we call such populations *subtly defined*. For instance, gamma-delta T cells, which are known for their antigen recognition capacities [11], are distinguished from ordinary cytotoxic T cells by the presence of just a few gamma and delta T receptors. Whereas PCA and ICA both compute features that optimize objective functions over the entire dataset―total variance and non-Gaussianity―rare cell populations thwart both strategies, since the genes defining them may be noisy or unexpressed over much of the data. Similarly, scVI uses the evidence lower bound (ELBO) loss function to evaluate and refine its latent encoding. Since ELBO takes each recorded transcript into account, rare and subtly defined cell types may not impact it much, leading to underrepresentation. Other non-linear methods, like UMAP [12] and t-SNE [13], rely on a *k*-nearest-neighbor graph; however, constructing an accurate *k*-nearest-neighbor graph requires an accurate notion of cell-cell similarity, which is nontrivial. The same can be said of network-based methods, like PHATE [9] and diffusion maps [10], which build reductions using diffusion over the *k*-nearest-neighbor graph. Real cellular populations may be both rare and subtly defined, so these challenges are significant roadblocks to realizing scRNA-seq’s full potential.

**Fig. 1:**
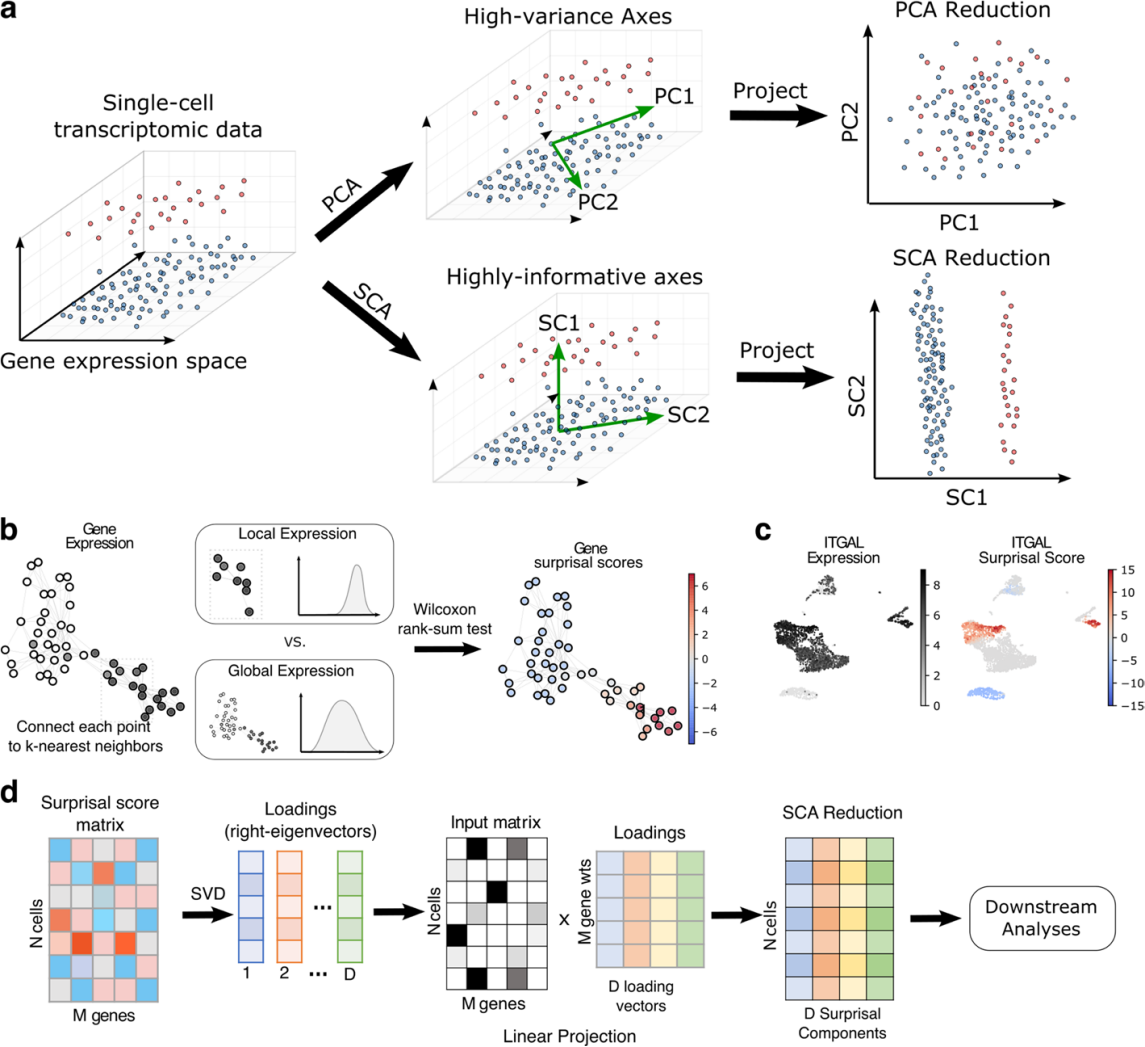
**a** Illustration of SCA’s key conceptual advance. The vertical axis separates a small cellular population (top) from a larger one (bottom). The two horizontal axes have higher variance but cannot separate the two populations. The leading principal components align with the higher-variance horizontal axes and fail to separate the populations. The leading surprisal component aligns with the more informative vertical axis, allowing downstream separation. **b** Construction of surprisal scores from gene expression data. For each cell, we compare the gene’s expression in a local neighborhood of the cell to the gene’s global expression using the Wilcoxon rank-sum test. The resulting *p*-values are negative log-transformed to give the “surprisal” of the observed over- or under-expression, and given a positive sign for over-expression and a negative sign for under-expression. **c** Surprisal scores of the *ITGAL* gene over a set of 3000 PBMCs profiled via Smart-seq 3 [14]. Scores are positive where the gene is locally enriched, near zero where it represents noise, and negative where it is conspicuously absent. **d** Construction of surprisal components. Surprisal scores undergo singular value decomposition (SVD) over all genes to yield *D* loading vectors that capture informative axes in the data. We then linearly project the input transcript count matrix to these axes, producing a *D*-dimensional representation of each cell for downstream analysis

Here, we introduce SCA (surprisal component analysis), an information-theoretic dimensionality reduction method that identifies statistically informative signals in single-cell transcriptional data to enable deeper insight into complex tissues (Fig. 1a). SCA newly leverages the notion of *surprisal*, whereby less probable events are more informative when they occur, to assign a *surprisal score* to each transcript in each cell. By identifying the set of axes which captures the most of this surprising variation, SCA enables dimensionality reduction that better preserves information from rare and subtly defined cell types, uncovering them where existing methods cannot.

To demonstrate the utility of our approach, we ran SCA on real and simulated data with rare and subtly defined cellular populations, and assessed our ability to recover these populations downstream. For comparison, we also tested PCA, ICA, scVI, diffusion maps, PHATE, and six rare cell type discovery tools: RaceID [15], GiniClust [16], CellSIUS [17], FiRE [18], GeoSketch [19], and Hopper [20]. We show that SCA enables detection of small populations, such as gamma-delta T cells and mucosal-associated invariant T (MAIT) cells, which are invisible to existing pipelines and yet critical to the study of tumor immunology [1, 2]. At the same time, SCA reductions better capture larger-scale differences between more common cell types, enabling multi-resolution analysis without the need for re-clustering [21]. Beyond rare cell type recovery, we found that SCA more accurately recovers gene-gene relationships and restores dropouts when used as a basis for MAGIC imputation [3].

SCA is highly efficient, requires no information aside from transcript counts, and generalizes to data comprised of discrete cell types or continuous trajectories. The output components have a clear linear relationship with the original transcripts, facilitating straightforward biological verification and interpretation. We believe that SCA’s information-theoretic approach is a mathematically justified and empirically useful approach to signal extraction in any high-dimensional data modality, biological or otherwise.

## Results

### Overview of SCA

Like PCA and ICA, SCA projects the input data to a linear subspace spanned by a set of basis vectors, which we call *surprisal components*. SCA’s key conceptual advance is its novel approach for finding *informative* axes of variation, where an informative axis is one that separates cell types or captures biologically meaningful variation (Fig. 1a). Single-cell experiments have shown that the presence or absence of a small number of genes can determine a cell’s phenotype [6, 22, 23]. The key challenge, then, is to find and isolate these signals for each cell.

To this end, SCA first quantifies the importance of each transcript in each cell by converting transcript counts into *surprisal scores* (Fig. 1b; Additional File 1: Algorithm 1). To determine the score of a given transcript in a given cell, we compare its expression distribution among the cell’s *k* nearest neighbors to its global expression (i.e., to the expected distribution of the transcript among a set of *k* cells randomly chosen from the entire dataset) for a user-specified neighborhood size *k*. A transcript whose local expression deviates strongly from its global expression is more likely to inform the cell’s location in relation to other cells, and therefore its identity. We quantify this deviation through a Wilcoxon rank-sum test, which produces a *p*-value representing the probability of the observed deviation in a random set of *k cells*. Following Shannon’s definition [24], the *surprisal* or *self-information* of the observed deviation is then defined as the negative logarithm of its probability, i.e., as $-\log (p)$. This is a positive number which measures how surprising the transcript’s local expression is, in units of nats when the logarithm is natural (changing the base scales the scores by a constant factor, which does not affect SCA’s output). To distinguish over- from under-expression, we flip the sign for under-expressed transcripts (Methods). The resulting scores are compiled into a *surprisal matrix* with the same dimensionality as the input data.

This strategy gives genes high positive scores where they are markers (genes that distinguish a cell type from the rest), scores near zero where they represent noise, and low negative scores where they are conspicuously absent (Fig. 1c). For example, consider a marker gene for a rare population. The gene is unexpressed over much of the data but highly expressed in cells belonging to the population. Thus, for these rare cells, the local expression is far higher than would be expected by chance, so the gene receives a high score for these cells. Likewise, a gene expressed everywhere *except* in a rare population receives low scores on members of the population. On the other hand, a noisy gene with no bearing on cellular identity receives low scores everywhere, since its distribution on *k*-neighborhoods resembles that of random sets of *k* cells.

We next sought to distill the signal captured by the surprisal matrix into a low-dimensional representation (Fig. 1d; Additional File 1: Algorithm 2). As shown in Methods and in Additional File 1: Supplementary Note 1, the right-eigenvectors of the surprisal matrix represent highly informative linear combinations of genes, which we call *surprisal components* (SCs). The first *D* right-eigenvectors, which we denote $v_1,...,v_D$, then span a linear subspace onto which we project the input matrix *X* of *N* cells by *M* genes. The resulting $N\times D$ matrix is the output of SCA. We emphasize that while the construction of the surprisal matrix and of $v_1,...,v_D$ is nonlinear, SCA’s output is a linear projection of its input to their span. This places SCA in the category of linear dimensionality reduction methods, together with PCA and ICA. This means that each of the output features is a weighted sum of genes, enabling straightforward interpretation. Furthermore, it enables many convenient workflows, including elbow plots (via the eigenvectors of the surprisal matrix, which we make readily available), and analysis of the gene loading vectors to interpret the components.

In summary, SCA accepts a transcript matrix and sets of *k*-nearest neighbors for each cell, finds transcripts that inform each cell’s locale, and distills this information into a smaller set of features. This process amplifies the locality signal of the input *k*-nearest neighbor data. For example, even if only 10% of a cell’s neighbors belong to the same population, this is still highly significant when the population comprises only 1% of the sample. SCA’s surprisal scores would reflect this, and the resulting components would better separate the rare cells, as we verify. The input neighborhoods can be specified arbitrarily, but by default SCA computes them via Euclidean distance on a PCA representation.

**Fig. 2:**
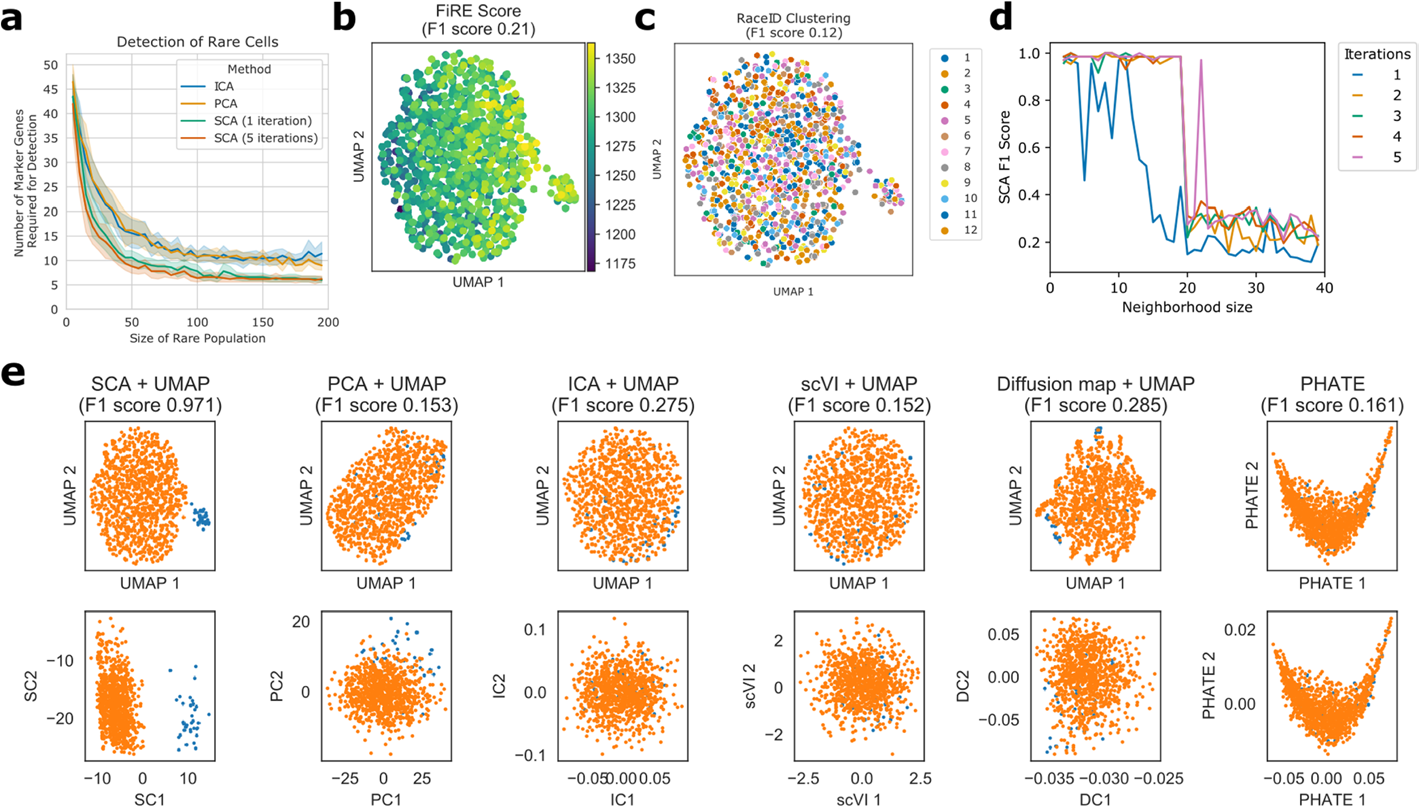
Performance of SCA on data simulated with Splatter [25]. **a** Ability of PCA, ICA, and SCA to recover rare cell populations of different sizes and with varying numbers of marker genes (out of 1000 cells and 1000 genes total). The population is considered “recovered” if the downstream Leiden clusters capture it with F1 score greater than 0.9 (Methods). SCA detects smaller populations with few marker genes. **b**, **c** Performance of FiRE [18] and RaceID [15] on a simulated dataset where 3% of the cells are defined by 10 marker genes (out of 1000 cells and 1000 genes total). For easy comparison with (e), FiRE scores and cluster memberships are plotted in the UMAP embedding downstream of the SCA representation. **d** Performance of SCA on the same dataset with a variety of neighborhood sizes. With a neighborhood size of 20 or fewer, SCA captures the rare population with very high F1 score after 2 or more iterations. The F1 score decreases when the neighborhood size approaches the size of the rare population, though it remains higher than PCA’s score of 0.153. **e** Top: UMAP plots downstream of various dimensionality reduction strategies, as well as the PHATE embedding [9]. SCA alone separates the rare population. Bottom: Scatter plots of the first two components of each reduction. The leading surprisal component separates the rare population from the rest

**Fig. 3:**
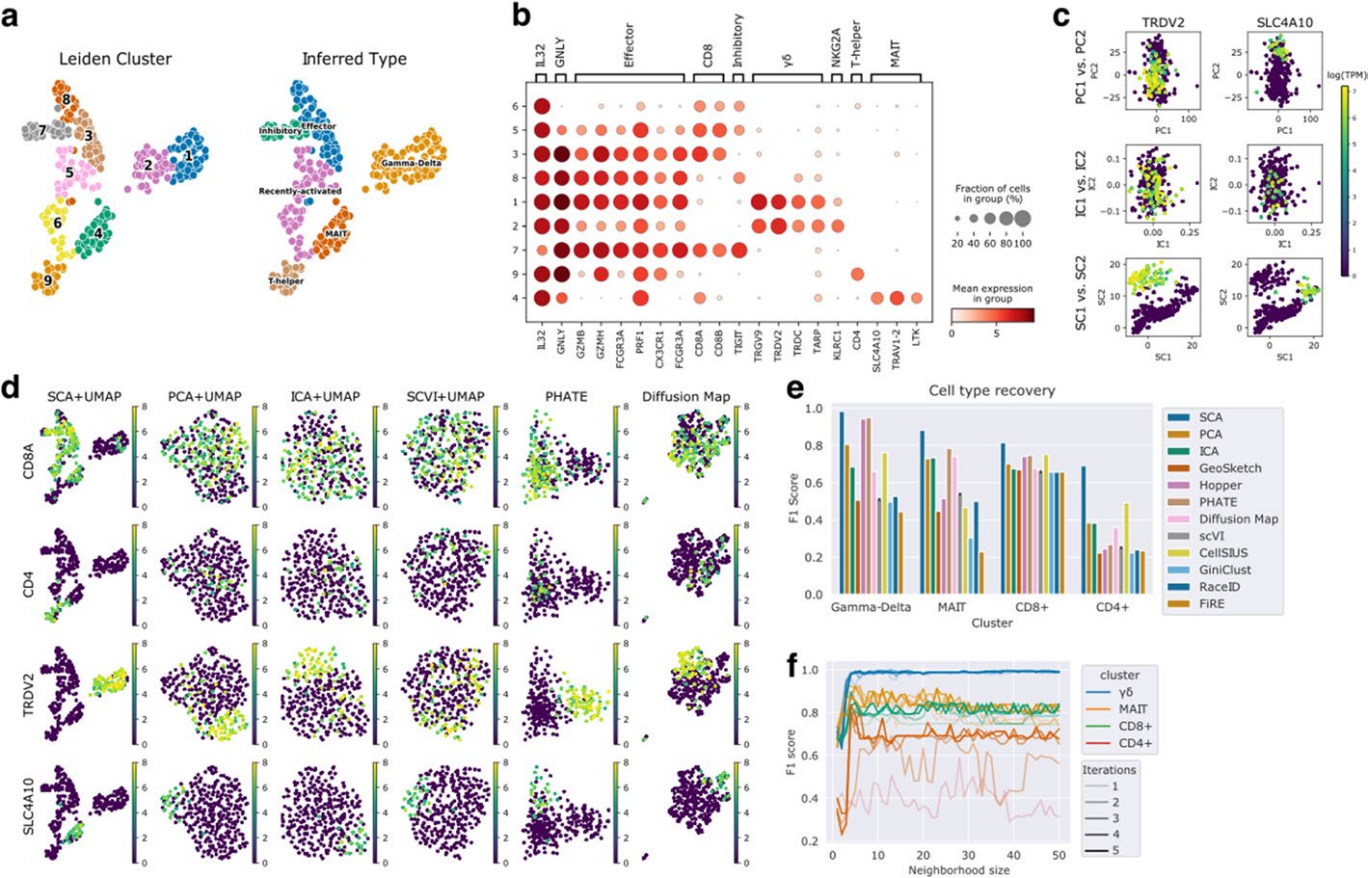
SCA recovers subtly defined cellular populations from a set of 307 cytotoxic T cells profiled using Smart-seq 3 [14]. **a** UMAP embedding computed from a 20-dimensional SCA representation using Euclidean nearest neighbors, with Leiden clusterings (left) and inferred cell types (right). Gamma-delta, MAIT, and T helper populations cleanly separate. **b** Dot plot of key marker gene groups in each SCA-derived Leiden cluster. Gamma-delta, MAIT, and T helper cells are clearly identifiable from their known marker genes. **c** Scatter plots of leading principal, independent, and surprisal components, colored by log-TPM (transcript per million) expression of key marker genes: the delta-receptor TRDV2 marks gamma-delta T cells [11], and SLC4A10 marks MAIT cells [26]. The leading surprisal components cleanly separate the gamma-delta and MAIT subpopulations, whereas the leading PCs and ICs blur these distinctions. **d** UMAP plots derived from 20-dimensional PCA, ICA, scVI, and SCA, and diffusion map embeddings of the data, as well as the PHATE embedding (Methods). CD8 T cells, CD4+ T helper cells, TRDV2+ gamma-delta T cells, and SLC4A10+ MAIT cells form distinct regions of the SCA-derived UMAP plot. **e** F1 scores for recovery of major T cell populations by various clustering schemes (Methods). For PCA, ICA, SCA, scVI, and diffusion maps, we assess Leiden clusters from the Euclidean 15-nearest neighbors graph with resolution 1. Leiden clusterings computed on the SCA representation consistently capture these cell types with highest accuracy. **f** Robustness analysis for cell type recovery with respect to the size of the neighborhoods used to compute SCA’s surprisal scores and the number of iterations. Performance improves with more iterations, and is stable across a wide range of neighborhood sizes

This signal-boosting step can be repeated: from an initial SCA reduction, we can compute *k*-neighborhoods using the Euclidean metric, use these neighborhoods to compute a surprisal matrix, and perform singular value decomposition to compute another SCA reduction. As we show for both real and Splatter-simulated data [25], this often improves the representation of rare and subtly defined cell types (Figs. 2d and 3f). Intuitively, we begin with a weak notion of locality (provided by PCA) and continually refine it. In our experiments, we have found that performance usually stabilizes after 3–5 iterations and remains stable thereafter (Fig. 3f, Additional file 1: Fig. S2). Note that regardless of the number of iterations, SCA’s output remains a linear projection of its input, since iteration simply refines the subspace onto which SCA projects.

### SCA recovers rare populations from noisy synthetic data

To test SCA’s power to uncover rare cell populations under a wide range of conditions, we used Splatter [25] to generate synthetic datasets with rare populations of various size and with varying numbers of marker genes. Each simulated dataset contains 1000 cells and 1000 genes, with 2–200 rare cells marked by 2–50 differentially expressed genes. The remaining simulation parameters, such as library size distribution, outlier gene probability, and gene expression distribution, were estimated by fitting Splatter to a widely used dataset of 3000 peripheral blood monocytes profiled by 10X genomics [27] (Methods).

To test the ability of SCA, ICA, and PCA to recover rare populations, for each combination of marker gene count and rare population size, we examined 10 replicate Splatter datasets generated with different random seeds. To assess population recovery, we computed Leiden clusterings from 20-dimensional embeddings using each method, chose the subset of Leiden clusters that best identified the rare population, and computed the F1 score (Methods). We consider the rare population recovered if this F1 score is 0.9 or greater.

We found that on average, SCA requires 38% fewer marker genes than PCA and 39% fewer marker genes than ICA to recover a rare population of a given size (Fig. 2a). Similarly, for a fixed number of marker genes, SCA can detect rare populations on average 29% smaller than those of PCA, and 28% smaller than those of ICA. For example, when the rare population contains 100 cells, SCA requires an average of 6.4 marker genes to detect it, whereas PCA and ICA require on average 10.8 and 10.6 marker genes, respectively. A *t*-test across the 10 replicates confirms that SCA consistently requires fewer marker genes than either PCA or ICA ($p<0.001$). Thus, SCA enables better recovery both of rare and of subtly defined populations.

To allow more comprehensive benchmarking and visualization, we used the same Splatter parameters to generate a dataset where each cell has a 2.5% chance of belonging to a rare population with 10 marker genes. Due to sampling noise, the resulting dataset had 34 rare cells (3.4% of the total). We performed dimensionality reduction on this dataset using PCA, ICA, SCA, scVI, diffusion maps, and PHATE, as well as several tailored methods for rare cell type discovery: CellSIUS ([17]), RaceID ([15]), GiniClust3 ([16]), and FiRE([18]) (Fig. 2b, c, e). To obtain an F1 score from FiRE, which assigns cells a continuous rarity score, we consider all sets containing the top *n* rarest cells for $1<n<1000$ and report the highest F1 score of any such set (Methods). As GiniClust3 did not identify any of the marker genes as having significantly high Gini index, it combined all cells into a single cluster. Similarly, CellSIUS did not identify any genes with a bimodal distribution and did not generate a clustering.

SCA cleanly separates the two populations (F1 score 0.971) whereas the other methods do not (F1 score $<0.3$; Fig. 2e). In UMAP plots downstream of each reduction, only SCA shows a clear separation (Fig. 2e, top row). The first surprisal component (SC) distinguishes the rare population, whereas the leading principal, independent, or diffusion components do not (Fig. 2e, bottom row). Thus, SCA better extracts features that recover rare populations, enabling more sensitive and specific detection.

We found that SCA’s performance improves with more iterations and is stable for neighborhood sizes ranging from 2 to 20 (Fig. 2d). At larger neighborhood sizes, the F1 score is comparable to that achieved by other methods. This behavior is expected: since large neighborhoods cannot be contained in very small populations, SCA has limited ability to identify populations with similar or smaller size than the chosen neighborhood size. We therefore recommend choosing a neighborhood size smaller than the expected size of the rarest cellular population in the sample. Our simulations (Figs.  2d and 3f) show strong cluster recovery even at neighborhood sizes smaller than 10, so this is not a substantial limitation. 

### SCA reveals the landscape of cytotoxic T cell subtypes

Novel therapies increasingly leverage the immune system to fight disease, and the complexity and cellular diversity of immunological tissues make them ideal targets for scRNA-seq [6, 23, 28]. However, these tissues also challenge the technology in a variety of ways: they contain diverse cell types with rare but clinically important sub-types, and the expression of individual surface receptors has outsize effects on phenotype, leading to many subtly defined cell types [23, 28]. We therefore examined whether SCA can find and distill these signals to reveal a richer landscape of immune cell types.

We obtained a collection of 307 cytotoxic T cells profiled using Smart-seq 3 (SS3) [14]. After standard log transcript per million pre-processing with a pseudocount of 1 (Methods), we computed 20-dimensional reductions using PCA, ICA, SCA, scVI, and a diffusion map. We ran SCA with 1-5 iterations and the default neighborhood size of 15. We followed each reduction with standard downstream steps: 15-nearest neighbor graph construction using the Euclidean metric, UMAP embedding, and Leiden clustering. In addition, we computed a two-dimensional embedding using PHATE and a clustering using PHATE’s own clustering function based on internally computed similarities.

The PCA, ICA, and scVI reductions suggest that the data is homogeneous: the UMAP visualizations are globular, with no distinct clusters, and there is no obvious structure in the leading components (Fig. 3c and d). On the other hand, SCA’s embedding reveals several clearly separated populations, summarized by 9 Leiden clusters (Fig. 3a). Differential gene expression analysis shows that these correspond to known cell types (Fig. 3b). For example, clusters 1 and 2 contain Gamma-delta T cells, as indicated by expression of the gamma- and delta-receptors *TRGV9* and *TRDV2* [11]. Cluster 4 expresses *SLC4A10*, *TRAV1-2*, and *LTK*, strongly suggesting that this cluster contains MAIT cells [2, 26]. CD4+ T helper cells group neatly in cluster 9, whereas high *TIGIT* levels in cluster 7 suggest an inhibitory phenotype [29, 30]. Clusters 3 and 8 express standard cytotoxic effector genes like granzymes and perforins, whereas clusters 5 and 6 express CD8 but have low granzyme expression, suggesting recently activated CD8 T cells.

We further tested the ability of each method to detect key immunological marker genes (Fig. 3d). The UMAP plots derived from the PCA, ICA, scVI, PHATE, and diffusion map representations do not separate cells based on key immunological markers, whereas the SCA-derived UMAP plot does. To quantify this finding and see how it affects de novo population discovery, we assessed whether the Leiden clusters computed downstream of each representation were concordant with a marker-based classification. We defined CD8+ T cells as those expressing *CD8A*; CD4+ T cells as those expressing *CD4*; gamma-delta T cells as those expressing at least two of *TRGV9*, *TRDV2*, and *TRDC*; and MAIT cells as those expressing at least two of *SLC4A10*, *TRAV1-2*, and *LTK*. We computed F1 scores for the recovery of each of these types as described for synthetic data and in Methods. We did the same for clusterings output by CellSIUS, RaceID, GiniClust, GeoSketch, and Hopper. To produce clusterings using Hopper, we performed Leiden-clustering on a 50-point Hopper sketch of the data, then assigned each cell the cluster label of its nearest sub-sampled cell. For GeoSketch, we projected the data to 20-dimensional PCA coordinates computed from a 50-point sketch, and then computed Leiden clusters. SCA consistently outperforms the other methods in identifying these immunological classes (Fig. 3e). As with the synthetic data, we found that the leading surprisal components distinguish cell types, whereas leading independent and principal components do not (Fig. 3c). Thus, SCA is better-suited to detect subtly defined immune cell types.

We next performed robustness analysis to see how SCA’s performance varies under different choices of neighborhood size *k* and different numbers of iterations (Fig. 3f). As with the synthetic datasets, running more iterations often improves the representation; notably, the CD4+ sub-type is not consistently clearly captured until at least the third iteration. After 3, 4, or 5 iterations, SCA performs well on all sub-types over a wide range of neighborhood sizes (from 5 to at least 90). Very small neighborhood sizes ($<5$) do not perform as well, likely due to a lack of statistical power. On the other hand, results on simulated data suggest limited ability to recover cellular populations with size similar to or smaller than the neighborhood size (Fig. 2d). For most datasets, this leads to a wide span of acceptable choices of neighborhood size. We also found that SCA’s performance is robust to the number of components in the reduction, performing well when as few as 5 and as many as 50 components are taken (Additional File 1: Fig. S6c).

While single-cell analysis pipelines frequently subset to highly variable genes (HVG) to remove noisy or lowly expressed transcripts, marker genes for small populations intrinsically have low variance. To ensure that these were not removed in the above experiments, we did not perform highly variable gene (HVG) selection prior to dimensionality reduction. To test the effect of HVG subsetting, we repeated all of these experiments on the same dataset after filtering to the 1000 most variable genes and log transcript per million preprocessing (Additional File 1: Supplementary Note 6; Additional File 1: Fig. S6a,b). We found that SCA performs substantially better when all genes are kept, and outperforms the other tested methods regardless of whether gene filtering is performed.

### SCA distinguishes known cell types profiled by CITE-seq

To evaluate whether SCA’s reductions better detect known populations of cells in a larger-scale immmunological single-cell dataset, we obtained a CITE-seq dataset from Hao et al. [6], in which hundreds of thousands of PBMCs from 8 human donors were subjected in parallel to transcriptomic profiling and to surface receptor profiling with a panel of 228 antibodies. The authors use both modalities, and input from human experts, to produce a cell type classification, which we take as a ground truth. We subsetted the data to T cells, yielding 73,000 cells across all donors, and assess performance in each donor individually. For each donor, we computed 50-dimensional representations using PCA, ICA, SCA, scVI, and a diffusion map. We also compute 2-dimensional PHATE embeddings, and clusterings using PHATE’s built-in function.

**Fig. 4:**
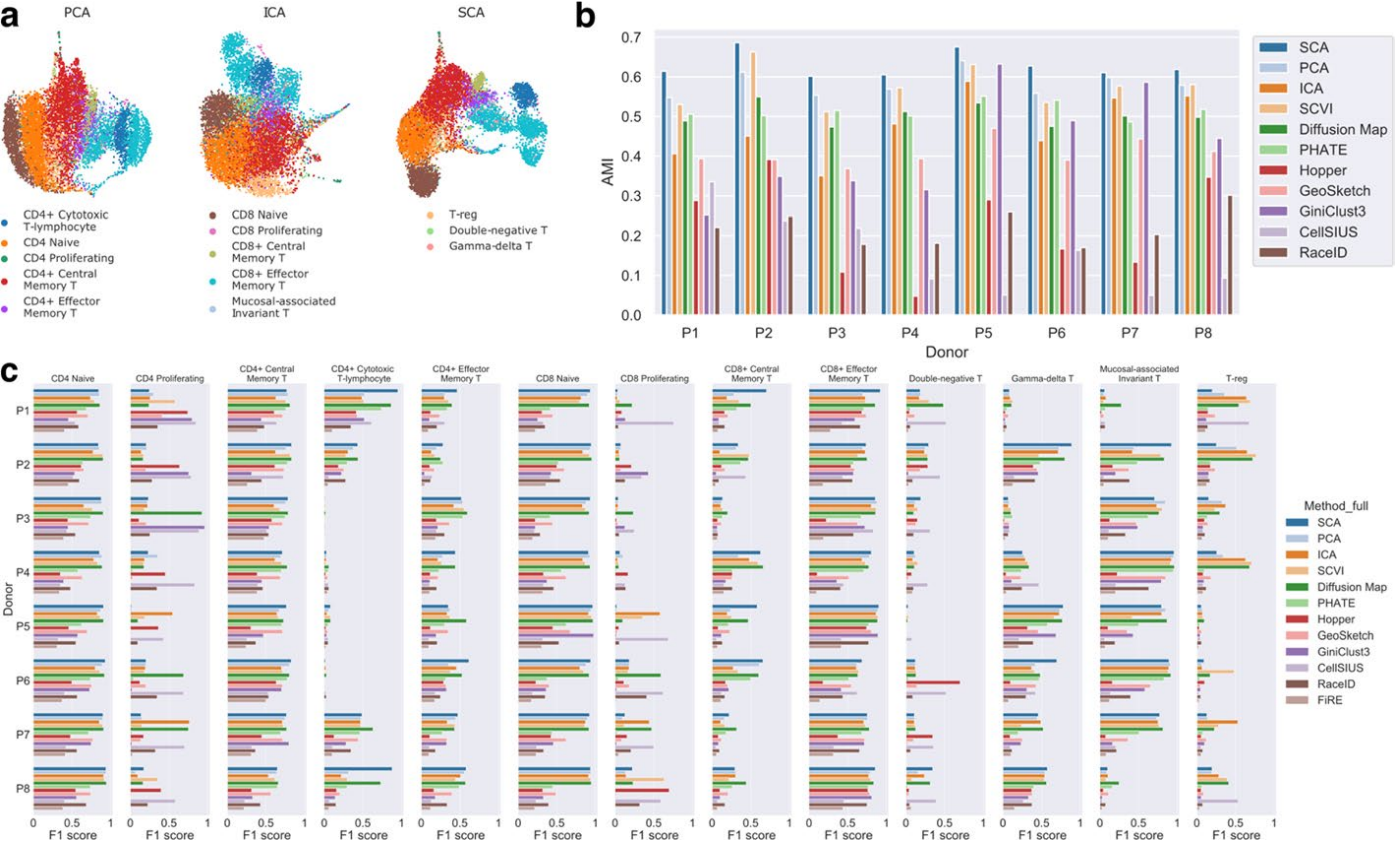
SCA outperforms many other methods, both general and problem-specific, at rare and subtly defined cell type discovery on a large PBMC dataset with ground-truth cell labels. **a** UMAP plots computed from PCA, ICA, and SCA reductions of T cell scRNA-seq data for patient 1 from the Hao et al. [6] dataset. Cell type labels were determined by the original authors via parallel screening of 228 antibodies using CITE-seq. **b** Adjusted mutual information (AMI) of true cell labels with clusters output by each of the 11 methods tested in each patient (FiRE does not output a clustering but a rareness score and thus is not amenable to AMI analysis). For PCA, ICA, SCA, and scVI, we perform Leiden clustering with resolution 1.0 after reduction. SCA-based clusterings consistently have higher AMI with the true labels. **c** F1 scores for recovery of all T cell subtypes across all 8 patients of the dataset from Hao et al., from PCA, ICA, and SCA followed by Leiden clustering with resolution 1.0, and from nine other methods. For each clustering and cell type, the set of clusters best identifying that cell type was selected, and the F1 score reported

Compared to PCA and ICA, SCA consistently generates more structured UMAP plots downstream, with clearer visual separation between cell types. UMAP plots computed from PCA, ICA, and SCA are shown for patient 1 in Fig. 4a, and similar UMAP plots for the remaining patients and reductions are provided in Additional file 1: Fig. S4. We observe similar improvements when plotting the first two components of each reduction against each other (Additional file 1: Fig. S5). We hypothesized that this separation leads to more accurate clusterings downstream. To test this theory, we performed Leiden clustering [31] on each representation with resolution 1.0 and computed the adjusted mutual information (AMI) between the Leiden clusters and the true cell labels [32]. For further comparison, we also computed AMIs for Leiden clusterings computed from GeoSketch and Hopper reductions, and clusterings returned by GiniClust3 [16], CellSIUS [17], and RaceID [15]. Across all patients, the SCA-derived Leiden clusterings achieved the highest AMI with the true cell labels (Fig. 4b). Thus, SCA representations enable more accurate downstream recovery of cellular populations in a sample.

To determine the accuracy with which SCA and other methods detect each of the known cellular populations, we used the F1 score measure (Methods). Across all donors, we found that SCA consistently ranks among the highest F1 scores, with some cell types recoverable only by SCA (Fig. 4c). For example, in patient 1, SCA recovers CD4 cytotoxic T lymphocytes (CD4 CTL), a subtly defined population distinguished from CD8 CTLs mainly by the presence of CD4, with F1 score 0.97. As notable exceptions, CellSIUS and GiniClust perform exceptionally well on the CD4 proliferating population, with CellSIUS also outperforming other methods on the CD8 proliferating and double-negative T populations. We suspect that the Leiden clusterings under-perform here due to theoretical limitations of community-detection algorithms to detect very small populations, as discussed in Kumpula et al. [33].

### SCA improves graph-based imputation

Dropouts and technical noise often obscure gene-gene relationships in single-cell data. Imputation aims to recover lost transcripts and restore these relationships. MAGIC [3] tackles imputation by constructing a diffusion operator to share information across similar cells, achieving better recovery of gene-gene interactions than a variety of other methods, including simple *k*-NN imputation, low-rank approximation, and smoothing based on diffusion components [10, 34, 35]. By default, MAGIC computes cellular similarity using the Euclidean distance in PCA space. Since SCA better separates biological cell types, we pursued the intuition that using Euclidean distance in an SCA reduction would allow MAGIC to build a better diffusion operator, yielding more accurate imputation. We therefore formulated *SCA-MAGIC*, which performs diffusion over an SCA embedding instead of a PCA embedding (Methods).

**Fig. 5:**
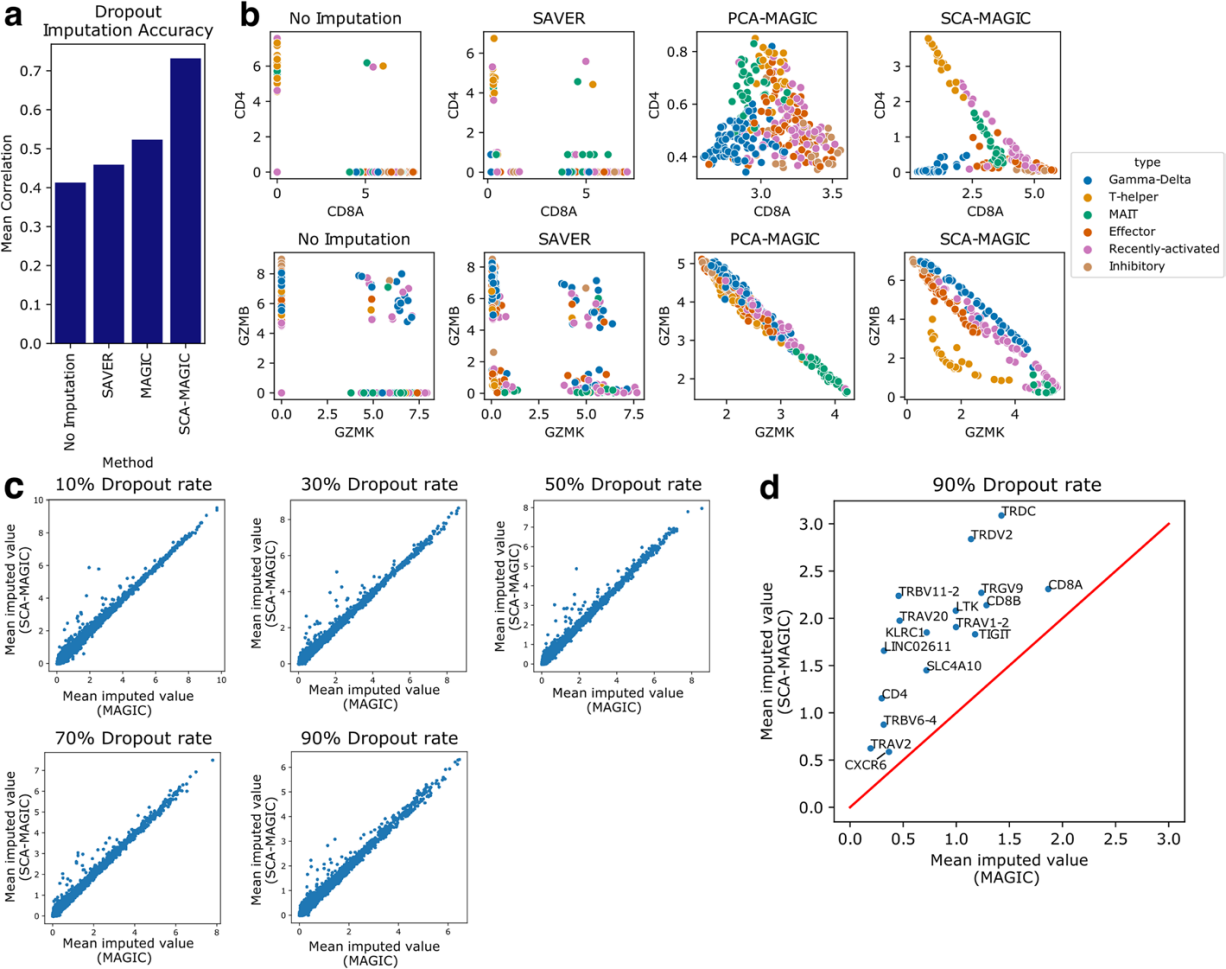
Imputation performance using SAVER, MAGIC and our SCA-MAGIC. **a** Recovery of marker genes on the Splatter dataset analyzed in Fig. 2b–e. For each method, we measure the average correlation between the marker genes after imputation and an indicator vector for the rare cells. MAGIC achieves significantly higher correlation using SCA as a base embedding (SCA-MAGIC). **b** Visualizing gene-gene relationships in cytotoxic T cell data after imputation using SAVER, MAGIC or our SCA-MAGIC. SCA-MAGIC better recovers the inverse relationships between CD8 and CD4 and between granzyme B and granzyme K, with gamma-delta T cells expressing neither CD8 nor CD4 and lower granzyme expression for T helper cells. **c** Scatter plot showing dropout recovery in the cytotoxic T cell dataset at various dropout rates. A fixed percentage of nonzero transcript measurements were set to zero, and the mean imputed values of these removed transcripts were assessed for each gene. While MAGIC and SCA-MAGIC perform similarly on most genes, SCA-MAGIC consistently performs better on a subset of them, measured by dropout rate. **d** A closer look at the genes where SCA-MAGIC significantly outperforms MAGIC in recovering dropouts at the 90% dropout rate. They include many key marker genes such as *CD8A*, *CD8B*, *CD4*, emphTIGIT, and *TRDV2*

To test the ability of each method to recover dropouts from the 1000-cell Splatter synthetic dataset analyzed in Fig. 2b–e, we performed imputation using MAGIC and SCA-MAGIC and measured the Pearson correlation between the imputed values of the marker genes and an artificial “ground-truth” marker gene expressing 1 transcript on each rare cell and 0 transcripts elsewhere (due to normalization, the number of transcripts expressed in the rare population does not affect the Pearson correlation). We also tested SAVER [36], another imputation approach that uses regression instead of diffusion. As shown in Fig. 5a, SCA-MAGIC achieves the highest correlation between imputed marker genes and the ground-truth marker gene (correlation $\sim 0.7$).

To assess recovery of gene-gene relationships, we ran MAGIC and SCA-MAGIC on the cytotoxic T cells from [14]. We find that SCA-MAGIC best recovers the complementary relationship between CD8 expression and CD4 expression among alpha-beta T cells, with T helper cells having high CD4 levels and gamma-delta T cells expressing neither surface receptor, consistent with the literature [11, 37] (Fig. 5b, top). Both MAGIC and SCA-MAGIC report an inverse relationship between the expression levels of granzyme B and granzyme K. However, SCA-based MAGIC assigns the T helper cells lower granzyme B levels than the other populations. This finding is concordant with flow-cytometry results, which indicate that CD8+ T cells are the primary secretors of granzyme B [38]. SAVER-imputed data does not show smooth correlations and resembles the raw data.

To compare dropout recovery of MAGIC and SCA-MAGIC on real data, we created low-coverage versions of the cytotoxic T cell dataset by setting 10%, 30%, 50%, or 90% of the nonzero transcript counts to zero and examined the values of these dropped-out transcripts after imputation. Higher imputed values indicate better transcript recovery. For most genes, SCA-MAGIC and MAGIC perform similarly (Fig. 5c). However, SCA-MAGIC outperforms MAGIC on a small set of key marker genes, including *CD8A*, *CD8B*, *CD4*, *TIGIT*, *KLRC1*, and the gamma-delta marker genes *TRDV2* and *TRGV9* (Fig. 5d). Since these genes define important T cell subclasses, this improvement is consequential.

The creation of false positive signals is a concern in imputation [39]. We found that SCA-MAGIC performed at least as well as MAGIC at avoiding creation of false positive gene counts and artificial marker genes (Additional File 1: Fig. S7).

### SCA scales to large datasets

As improving technologies generate ever larger datasets, the computational tools used to analyze these datasets must scale accordingly. SCA meets this need with fast runtimes and modest memory overhead. Asymptotically, SCA’s runtime and memory overhead are both linear in the size of the input dataset (Additional File 1: Supplementary Note 2; Additional File 1: Fig. S1). To test this empirically, we measured runtime and peak memory usage for one iteration of SCA on data of varying sizes (Fig. S1). We produced test datasets by taking random subsets of patient 1’s T cell data from Hao et al. [6] with varying numbers of cells and genes. For all tests, we used a neighborhood size of 15. As expected, we find SCA’s runtime and memory performance *scale linearly* with increasing numbers of cells. On a subset with 9000 cells and over 20,000 genes, SCA takes 3 min and 15 s to run with a peak allocation of 561 MB. This is only slightly slower than PCA, which finishes in 2 min and 10 s and allocates 179 MB (ICA is somewhat more computationally demanding, requiring 266 s and allocating over 4GB). SCA’s linear scaling makes it tractable even on the very largest single-cell datasets; for example, on a mouse brain dataset from Saunders et al. with 939,489 cells and 20,658 genes [4], SCA runs in 5.5 h and allocates a maximum of about 29 GB, well within the range of most modern day computers. Sub-sampling techniques that preserve rare cell types, such as Hopper [20] and Geosketch [19], may be combined with SCA to enable fine-grained analyses of these massive datasets even on a laptop. We can reduce the memory overhead further by processing the data in chunks containing a user-specified number of genes (Methods). Chunk size is implemented as a parameter in the core function of SCA’s Python package.

### SCA performs well in the presence of batch effects

**Fig. 6:**
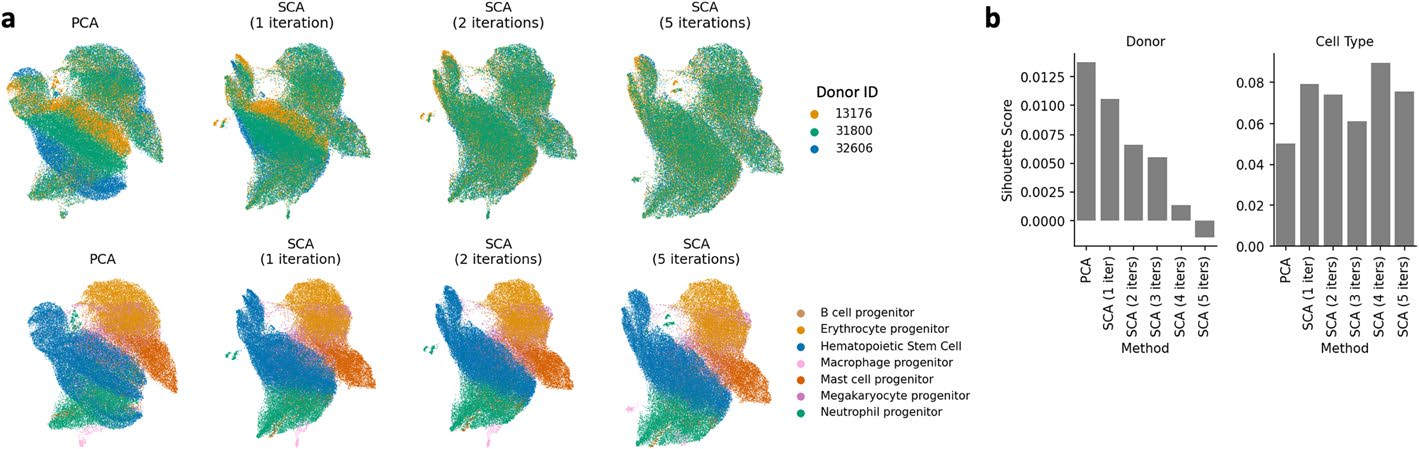
**a** UMAP plots downstream of PCA and SCA on CD34+ immune cell data with 7 cell types and 3 donors. With further iterations, batches appear more integrated (top) whereas cell types remain separate (bottom). **b** Silhouette scores of the donor and cell type groupings, using Euclidean distance downstream of PCA or SCA. The donor silhouette score decreases with further iterations of SCA (left), whereas the cell type silhouette score remains above that of PCA (right)

Single-cell experiments often include data from different samples, and sometimes different donors, introducing unwanted technical variation known as batch effects. While SCA is not designed to remove batch effects, we aimed to verify that its signal-boosting procedure does not emphasize them at the expense of true biological variation. To this end, we obtained scRNA-seq data comprising approximately 70,000 CD34+ cells from 3 human donors [40], combined the data without performing batch correction, computed PCA and SCA reductions with 20 components each, and assessed the impact of donor on each reduction. UMAP plots downstream of SCA showed significantly more overlap between donors than those downstream of PCA, and performing more iterations of SCA increased the degree of overlap between donors while keeping biological cell type separate (Fig. 6a). To quantify this finding, we used the silhouette score [41] to measure separation between donors and between cell types. The donor silhouette score is lower when using SCA, and decreases with more iterations, indicating better batch integration (Fig. 6b). By contrast, the cell type silhouette score was higher for SCA than for PCA, consistent with our other results. Thus, SCA’s reductions emphasize cell type-related biological differences over technical batch effects.

## Discussion

SCA offers an information-theoretic approach to measuring and extracting salient transcriptional signals in single-cell data, enabling downstream analyses at unprecedented resolution. By iteratively boosting the locality-specific signal of individual transcripts, SCA uncovers clinically relevant immunological populations that are invisible to existing approaches.

A variety of approaches have arisen that are specifically tailored to the problem of rare cell type recovery. However, we find that these methods have limiting assumptions [16, 18] or rely on potentially inaccurate or ill-defined clustering procedures [15, 17] that limit performance. GiniClust [16] assumes that genes with high Gini index are the most important; yet, we demonstrated that this is not always the case (e.g., the marker genes in the synthetic dataset were not marked as having high-Gini index by the algorithm). RaceID and CellSIUS both compute initial clusterings which are then individually refined. However, an accurate initial clustering may be difficult to obtain when cell types of interest are rare or subtly defined, and cluster-based approaches are unsuitable when cells form more continuous transcriptional structures, such as developmental trajectories, which do not neatly partition [42]. On the other hand, FiRE [18] sidesteps the limitations of clustering by assigning each cell a rarity score according to its degree of isolation, but the notion of isolation in turn relies on a meaningful cell-to-cell distance metric, which is not readily derived. Hopper [20] reduces the data in the hopes of increasing the proportion of rare cells, but its approach requires a reliable distance metric and requires discarding observations. For the latter two methods, one might improve performance by using Euclidean distance in an SCA representation as a distance metric. Our work suggests that the right dimensionality reduction can enable recovery of even rare and subtly defined populations.

SCA’s surprisal scores are similar in principle to the inverse document frequency (IDF) transform, a normalization approach widely used in text processing and in some single-cell applications, whereby each feature (gene) is weighted by the logarithm of its inverse frequency [43]. Like SCA, IDF gives rarely seen features more weight; however, it does not consider the locality-specific context of each feature measurement, so it lacks the statistical power to detect locally enriched signals. By incorporating counts from local neighborhoods of each cell, SCA allows genes to have variable scores across the dataset, achieving high-magnitude scores where they are discriminative and near-zero scores where they are noise (Fig. 1c). Our approach is designed to reflect true biology, where genes may be expressed sporadically across the entire dataset but mark informative distinctions only within a small subpopulation.

SCA is also conceptually similar to surprisal analysis [44], which compares observed data to a pre-computed balance state to identify meaningful deviations. Originally developed for thermodynamics, these methods have recently found use in *bulk* transcriptomic analysis of biological systems in flux, such as cancer cells undergoing epithelial-to-mesenchymal transition and carcinogenesis [45–48]. For example, Gross et al. [45] perform singular value decomposition on a surprisal matrix derived from time series micro-arrays to identify bulk transcriptomic signatures that predict eventual malignancy. In their work, the surprisal of a transcript is defined by the negative logarithmic fold change of the transcript from its value in the balance state. We attempted to generalize this idea to single-cell data by treating each cell as a separate time point, and computing surprisals as negative log-fold changes between observed transcript counts and transcript expression means across all cells. However, we show this extended notion of surprisal is under-powered and inaccurate for single-cell data (Additional File 1: Supplementary Note 5; Additional File 1: Fig. S6), because individual transcript counts are themselves noisy. For example, on the cytotoxic T cell dataset, this approach fails to separate CD8 from CD4 T cells (Fig. S6). SCA’s novel approach of testing expression in *neighborhoods* of cells instead of individual cells lends statistical power and limits the impact of noise and dropouts, especially in combination with the robust Wilcoxon test.

Data visualization, which features prominently in many single-cell pipelines [12, 49], differs from dimensionality reduction, on which we focus. Whereas visualization aims to produce a two-dimensional rendering of the data, dimensionality reduction produces a smaller, but still many-dimensional representation which is then analyzed further downstream. Thus, data visualization tools complement dimensionality reduction rather than substitute for it. Indeed, visualizations are often built on dimensionality-reduced data; for example, UMAP plots in existing literature are often computed on PCA or ICA reductions [4, 50]. SCA complements existing visualization tools to facilitate exploratory analysis (Figs. 4a, 3a, and Additional file 1: Fig. S4).

SCA also combines well with *sketching* techniques, such as Geosketch [19] and Hopper [20], which generate subsamples of cells that retain transcriptional diversity. In turn, these sketching techniques rely on a low-dimensional representation of cells, which SCA may provide. As motivation for the latter, we have shown that SCA is better at identifying rare cell types than these sketching techniques.

Although the process that generates the surprisal components is nonlinear, requiring nearest-neighbor graphs and Wilcoxon score computation, SCA’s output is a linear projection of its input. This places SCA firmly in the linear category, together with PCA and ICA; indeed, for fixed dimension *D*, the coordinate systems defined by SCA and by these methods are related by rotation in the original high-dimensional space. Intuitively, SCA changes the “perspective” from which the data is viewed. It is remarkable, then, that SCA’s reductions look so different in downstream analyses from those of PCA and ICA (e.g., Fig. 3a). This observation is possibly because high-dimensional space offers a far wider variety of perspectives than the three-dimensional space we often think in, giving linear methods more richness than they are usually credited for.

## Conclusion

Dimensionality reduction addresses the underlying goal of nearly all single-cell analytic pipelines―to determine which cells are phenotypically similar to one another or, in mathematical terms, to derive a biologically meaningful metric between cells. If we could meet this goal perfectly, we could immediately obtain perfect clusterings of single-cell data (each cluster would be a connected component of the *k*-nearest neighbor graph), perform perfect batch correction (by integrating cells based on phenotypic similarity), and substantially improve trajectory inference (by connecting similar cells along a continuous path). Dimensionality reduction represents single-cell data in a lower-dimensional Euclidean space, which inherits natural metrics (e.g., the standard Euclidean distance). Using information theory, SCA provides an embedding where Euclidean distance better captures biological similarity, causing cells with similar phenotypes to cluster together.

## Methods

### Details of the SCA Algorithm

#### Surprisal matrix computation (Additional File 1: Algorithm 1).

Given the input data *X* with *N* cells and *M* genes, a target dimensionality *D*, and a neighborhood size *k*, we first compute a *D*-dimensional PCA reduction of *X*. Using Euclidean distance in this PCA space, we compute for each cell *c* a neighborhood $N_k(c)$ containing the *k* nearest cells. Alternatively, the user may specify neighborhoods manually as lists of indices.

For each gene *g* and cell *c*, we then assess the significance of *g*’s expression in $N_k(c)$ as compared to its global expression. Under the null hypothesis, where *g* is randomly expressed, the local distribution $N_k(c)$ should be similar to the global expression. Using a Wilcoxon rank-sum test, we obtain a two-sided *p*-value $p_{c,g}$ representing the probability of the observed difference under this null hypothesis. We also offer two alternative *p*-values based on different models: a *t*-test, and a binomial test using only the binarized counts. We strongly recommend the Wilcoxon model for its flexibility to a wide range of data distributions, and robustness to different pre-processing protocols.

Small *p*-values indicate very unlikely events under the null hypothesis, leading to high surprisal. However, since tens of thousands of genes are often measured for each cell, we would expect $p_{c,g}$ to be very low for some cell-gene combinations even in the absence of true biological signal. We therefore adjust for multiple-testing within each cell using a family-wise error rate correction. If we assume that genes are uncorrelated, this correction takes the form

$$\begin{aligned} \tilde{p}_{c,g}=1-(1-p_{c,g})^{M} \end{aligned}$$

where *M* is the number of genes. The corrected value $\tilde{p}_{c,g}$ represents the probability that *any* gene has the observed deviation from the null distribution in *c*’s neighborhood. However, in real single-cell data, genes are often highly correlated, so the effective number of independent features is far fewer than *M*. As detailed in Additional File 1: Supplementary Note 4 and Additional File 1: Algorithm 3, we can identify a reasonable exponent $N_t$ by sampling many random sets of *k* cells from *X*, computing *p*-values from these random neighborhoods, and observing the distribution of these *p*-values. This provides a background model for contextualizing the $p_{c,g}$ values computed from the actual locally derived *k*-nearest neighborhoods and leads to the correction

$$\begin{aligned} \tilde{p}_{c,g}=1-(1-p_{c,g})^{N_t} \end{aligned}$$

where $N_t$ is often far less than *M*. When $N_t$ is computed as in Additional File 1: Supplementary Note 4, SCA does not produce erroneous clusters on negative control datasets which lack intrinsic structure, and randomly generated neighborhoods yield scores clustered around zero (Additional File 1: Supplementary Note 3; Additional File 1: Fig. S3). SCA computes $N_t$ in this way by default; however, users may also manually define $N_t$ to adjust the balance between sensitivity and specificity.

We next convert the corrected *p*-values $\tilde{p}_{c,g}$ into surprisal scores *I*(*c*, *g*). Shannon [24] defines the *surprisal* or *self-information* of an event with probability *p* as $-\log (p)$. Intuitively, less probable events are more informative when they occur. For a given cell *c* and gene *g*, $\tilde{p_{c,g}}$ is the probability of the event that that one of *c*’s genes has a local distribution at least as extreme as the observed distribution of *g*, under the null hypothesis of random gene expression. Thus, $-\log (\tilde{p_{c,g}})$ is the surprisal of this event and defines the magnitude of *I*(*c*, *g*).

To distinguish over-expression from under-expression, we give *I*(*c*, *g*) a positive sign if *g* is over-expressed in *c*’s neighborhood and a negative sign if it is under-expressed. Under the Wilcoxon model, over- or under-expression is determined by the sum of the ranks of *g*’s values in the *k*-neighborhood of *c* among all values *g* takes, which we denote $\text {ranksum}(g, N_k(c))$. Under the null hypothesis, this quantity follows a normal distribution with mean $\frac{k(N-k)}{2}$. Thus, we obtain

$$\begin{aligned} I(c,g)=-\text {sgn}\left( \text {ranksum}(g, N_k(c))-\frac{k(N-k)}{2}\right) \log (\tilde{p}_{c,g}). \end{aligned}$$

#### Computing surprisal components

From the surprisal scores *I*(*c*, *g*), SCA next seeks to generate an informative linear combination of genes. For a given combination defined by

$$\begin{aligned} \tilde{g} = \alpha _1g_1+\alpha _2g_2+...\alpha _Mg_M \end{aligned}$$

we say that $\tilde{g}$ has *loadings* $\alpha _1,...,\alpha _n$. For a fixed cell *c*, we formulate the surprisal score of $\tilde{g}$ as

$$\begin{aligned} I(c, \tilde{g}) = \alpha _1I(c, g_1)+\alpha _2I(c,g_2)+...+\alpha _MI(c,g_M) \end{aligned}$$

We then define the overall overall surprisal score of $\tilde{g}$ by taking the norm over all cells:

$$\begin{aligned} I(\tilde{g}) = ||\langle I(c_1,\tilde{g}), I(c_2, \tilde{g}),...,I(c_N, \tilde{g})\rangle || \end{aligned}$$

or, in matrix notation,

$$\begin{aligned} I(\tilde{g}) = ||{\textbf {S}}\mathbf {\alpha }^T || \end{aligned}$$

where ${\textbf {S}}$ denotes the surprisal matrix and $\mathbf {\alpha }=\langle \alpha _1,...,\alpha _M\rangle$.

We now seek the metagene $\tilde{g}$, defined by the loadings $\alpha _1,...,\alpha _N$, that maximizes $I(\tilde{g})$. Since we can achieve arbitrarily large values of $I(\tilde{g})$ by scaling the coefficients, we constrain the loading coefficients to have norm 1, that is:

$$\begin{aligned} ||\langle \alpha _1,...,\alpha _M\rangle || = 1. \end{aligned}$$

It is a standard linear algebra result that this maximum is realized by the leading right-eigenvector of $\textbf{S}$ (proof in Additional File 1: Supplementary Note 1 and [51]). Thus, the first surprisal component loading vector is simply the first right eigenvector of ${\textbf {S}}$, which we denote $\mathbf {v_1}$. To obtain additional surprisal components, we repeat the optimization with the constraint

$$\begin{aligned} \mathbf {\alpha }\perp \mathbf {v_1}. \end{aligned}$$

It is straightforward to see (Additional File 1: Supplementary Note 1) that this yields the second principal component loading vector $\mathbf {v_2}$ of ${\textbf {S}}$. Continuing, we see that the loading vectors for surprisal components are simply the right eigenvectors of ${\textbf {S}}$.

SCA next computes the values of the first *D* surprisal components over the *input* data (not the surprisal scores). That is,

$$\begin{aligned} {\textbf {SC}}_i({\textbf {X}}) = {\textbf {X}}\textbf{v}_i^T. \end{aligned}$$

Note that the surprisal components are linear functions of the input data, despite the nonlinear construction of $\textbf{S}$. Although the loadings are computed on $\textbf{S}$, the values of the components are computed by applying these loadings to ${\textbf {X}}$.

If desired, we can now use the resulting *D*-dimensional representation of *X* to compute a Euclidean *k*-nearest neighbor graph, compute a new surprisal matrix from these neighborhoods, and perform SVD on this new matrix to produce another *D*-dimensional representation of *X*. This can be repeated arbitrarily many times, and often improves performance up to 3–4 iterations (Fig. 3f, Additional file 1: Fig. S2).

Formal pseudocode for this algorithm is provided in Additional File 1: Algorithm 2.

#### Time and memory optimizations

Computing the surprisal scores *I*(*c*, *g*) for all cells *c* and genes *g* requires *NM* Wilcoxon rank-sum tests. However, we can rapidly produce all of the rank-sum statistics with minimal memory overhead as follows:

1. Divide the genes into chunks of a user-specified size *C*, depending on memory constraints (default 1000). Let $G_1=\{g_1,...,g_C\}, G2=\{g_{C+1},....,g_{2C}\}$, and so on.
2. For each gene chunk $G_i$:
  - Subset *X* to genes in $G_i$, obtaining a reduced dataset $X_i$
  - rank each column of $X_i$ to obtain a rank matrix $R_i$
  - Multiply the neighborhood adjacency matrix *A* with $R_i$, yielding a rank-sum matrix over neighborhoods, denoted $S_i$, overwriting $R_i$
  - Convert these rank-sums into *p*-values under to the null model, overwriting $S_i$ with a *p*-value matrix $P_i$
  - Convert these *p*-values into surprisal scores, as described above and in Additional File 1: Algorithm 1, overwriting $P_i$ with surprisal scores $S_i$.
  - Sparsify $S_i$ and store it. ($S_i$ is frequently quite sparse).
3. Concatenate the matrices $S_i$ horizontally to obtain the surprisal matrix *S*.

Using this approach, we only need to compute ranks for each gene once, and we avoid storing dense matrices of size larger than $N\times C$. Since *A* has at most *k* nonzero elements per row, the sparse matrix multiplication in step 2c requires only *O*(*kNC*) time. The remaining steps are easily accomplished with vectorized functions from scipy [52] and numpy [53].

With these improvements, SCA is nearly as fast as ICA and PCA, and uses significantly less memory than ICA (Additional File 1; Fig. S1). We include more in-depth time and memory benchmarks in Additional File 1: Supplementary Note 2.

#### Generating F1 scores from clusterings

To assess the accuracy with which a set of *T* clusters $c_1,...,c_T$ recovers a known population *P*, we used the following procedure:

1. Rank all clusters by the degree of overlap with *P*, i.e., by $\frac{|c_i\cap P|}{|c_i|}$. Ties may be handled arbitrarily. Assume without loss of generality that the indexing $c_1,...,c_T$ ranks the clusters in this way. Let *S* be an empty set.
2. for *i* in 1,2,...,*T*:
  - Measure the F1 score of $S\cup c_i$ with respect to *P*.
  - If this F1 score is higher than that of *S*, add $c_i$ to *S*. Otherwise, stop and return the current F1 score of *S* with respect to *P*.
3. Return the F1 score of *S* (if not already returned above).

If the target population is a union of clusters, this procedure is guaranteed to find it and return an F1 score of 1; otherwise, it finds a set of clusters whose union approximates *P* and returns the F1 score of their union with respect to *P*.

#### Synthetic data experiments using splatter

The synthetic dataset analyzed in Fig. 2 was generated using Splatter [25]. All but two parameters were determined by fitting the PBMC dataset from [27]. The fitted parameters are listed below:

- Mean rate parameter: 13.5
- Mean shape parameter: 0.583
- Library size location parameter: 7.69
- Library size scale parameter: 0.412
- Outlier probability: 0.025
- Outlier location parameter: 4.761
- Outlier scale parameter: 1.037
- Biological Coefficient of Variation dispersion: 0.2825
- Biological Coefficient of Variation degrees of freedom: 30.37

The two remaining parameters are (1) the probability of a gene being differentially expressed between the two groups and (2) the probability that a cell belongs to the smaller of the two groups (the “rare” population). We test all combinations of these two parameters for rare cell fractions ranging from 5 cells (0.5%) to 200 cells (20%) and for marker gene probabilities ranging from 0.2 to 5% in increments of 0.2%. For each combination, we generate ten synthetic datasets with different random seeds and run PCA, ICA, and SCA to make 20-dimensional representations of each replicate, keeping up to 5 iterations for SCA. We then compute Leiden clusterings on the 15-nearest Euclidean neighbor graph of each representation, with the default resolution of 1.0. From these clusterings we compute F1 scores for recovery of the rare population as described just above.

To determine how many marker genes are required to recover a population of a specific size, we filtered to all trials with the given population size, and recorded the lowest number of marker genes in any trial with F1 score greater than 0.9. We performed this analysis separately for each random seed used to generate replicates, resulting in 10 marker gene percentage values for each combination of rare cell fraction and method. These values are plotted in Fig. 2a.

#### Cytotoxic T cell population discovery

We extracted all cytotoxic T cells from the dataset in [14] using the authors’ cell type annotations, obtaining 307 cells in total. For PCA, we used scanpy’s pca function with 20 components. For ICA, we used the FastICA function of sklearn [54, 55], again with 20 components. For SCA, we ran five iterations with 20 components each, starting with the PCA representation. We ran scVI with default parameters on the top 4000 most variable genes (we observed little difference in performance when running on all genes). 15-nearest neighbors graphs were computed in each representation using Euclidean distance, and the results were used to generate the UMAP plots in Fig. 3. We computed diffusion maps using the 15-nearest neighbor graph downstream of the PCA representation, keeping 20 components (eigenvalue analysis confirms that this is a reasonable number of components, with further components adding noise). We computed the PHATE representation using the default parameters of the package (100 PCs, 5-nearest neighbors). Leiden clusters in each representation were computed downstream of Euclidean 15-nearest neighbor graphs, with the default resolution of 1.0. For PHATE, we use the built-in phate.cluster.kmeans with k=5. Dotplots to show expression of key marker genes were generated using scanpy’s dotplot function.

#### T cell data from Hao et al.

We obtained transcriptomic data from the authors’ website at https://atlas.fredhutch.org/nygc/multimodal-pbmc/. The log-transformed count data was subset to T cells using the authors’ annotations, yielding 73,259 T cells and 20,729 genes across all 8 patients. We then split by patient into 8 donor-specific datasets. For each donor, we computed an SCA reduction using 50 components and 5 iterations, with a neighborhood size of 100 (a larger neighborhood size is appropriate for larger datasets; neighborhood size did not greatly affect performance). Fifty principal components were computed using scanpy’s pca function, and 50 independent components were computed with the FastICA implementation provided by scikit-learn [54, 55]. To ensure convergence of FastICA, we raised the maximum number of iterations to 500 from the default of 200. We ran scVI with default parameters (learning rate 0.001, 400 warmup epochs for KL divergence term) and a 50-dimensional latent embedding space to match the dimensionality of the PCA, ICA, and SCA embeddings. Diffusion maps were likewise computed in 50 dimensions. We computed 15-nearest-neighbor graphs in each representation using the Euclidean distance metric, then ran UMAP [12] and Leiden clustering [31] on the resulting neighborhood graphs. For Leiden clustering, we use the default resolution of 1.0. PHATE embedding and clustering was run with default parameters using the phate Python package [9].

#### Imputation using MAGIC

To create SCA-MAGIC, we used the graphtools package to build a diffusion operator based on a 20-dimensional SCA reduction, with default parameters inherited from MAGIC (*knn = 5*, *knn_max = 15*, *decay = 1*, *thresh = 0.0001*). We used the same parameters to construct an analogous operator from a 20-dimensional PCA embedding. We then build MAGIC instances from these two operators, with time parameter *t = 5*, and compare performance on various datasets.

To generate artificial dropouts in the cytotoxic T cell data, we replaced a random subset of the pooled nonzero transcript measurements with zeros, comprising either 10%, 30%, 50%, or 90% of the total nonzero measurements. After performing imputation, we re-examined the transcripts that had been eliminated and checked whether they had been restored. High imputed values indicate well-restored transcripts.

#### CD34+ immune cells for batch performance benchmarking

We downloaded the training data from the multimodal single-cell integration challenge [40], consisting of 70,988 cells from 3 donors. The data were pre-processed by the original authors using a log transcripts-per-million transformation; we applied no further processing and used the cell types from the original authors to compute silhouette scores and visualize the data. We performed SCA with a wilcoxon scoring model and the default neighborhood size of 15, reducing to 20 components.

### Supplementary information

**Additional file 1.** Contains proofs of key mathematical results, formal descriptions of the algorithms underlying SCA, and results of additional experiments testing SCA’s performance and robustness.

**Additional file 2.** Contains the review history.

## Data Availability

SCA is implemented in Python, installed on PyPi for ease of use, fully documented, and offers ready integration with scanpy, a popular scRNA processing framework. Its source code is available under the MIT open source license, and the latest version can be found at https://github.com/bendemeo/shannonca [56]. A stable release is also available on Zenodo (doi:10.5281/zenodo.7854155, [57]). API documentation, installation instructions, and vignettes can be found at https://shannonca.readthedocs.io. For convenience, links to these pages are compiled along with a brief description of the method at http://sca.csail.mit.edu/. The SMART-seq3 data containing cytotoxic T cells has been deposited by the original authors under ArrayExpress E-MTAB-8735 at the European Bioinformatics Institute [58]. The T cell data from Hao et. al. [6] is available through GEO (GEO: GSE128639, [59]). The CD34+ immune cell dataset used for batch performance benchmarking is available as training data for the online Kaggle competition at https://www.kaggle.com/competitions/open-problems-multimodal/data.
