# Supplementary material for: SCA: recovering single-cell heterogeneity through information-based dimensionality reduction
Source: Genome Biol. 2023 Aug 25;24:195. doi: 10.1186/s13059-023-02998-7 (PMC10464206; doi:10.1186/s13059-023-02998-7)
Supplement: Supplementary file 1 — Additional file 1. Contains proofs of key mathematical results, formal descriptions of the algorithms underlying SCA, and results of additional experiments testing SCA’s performance and robustness. [file 13059_2023_2998_MOESM1_ESM.pdf]

# Additional File 1: Supplementary Notes and Figures

Recovering Single-cell Heterogeneity through

Information-based Dimensionality Reduction

Benjamin DeMeo and Bonnie Berger

June 19, 2023

## Supplementary Note 1: Mathematical Formalization

The main text uses the following lemma to justify SCA:

**Lemma 1.** *If $X$ is an $n \times m$ matrix and $\alpha$ is an $m$-dimensional vector with unit $L_2$ norm, then $||X\alpha^T||$ is maximized when $\alpha$ is a principal right-eigenvector of $X$.*

The following proof is adapted from [51]:

*Proof.* Note that

$$||X\alpha^T||^2 = \langle X\alpha^T, X\alpha^T \rangle = \langle X^T X\alpha^T, \alpha^T \rangle.$$

Since $X^T X$ is symmetric, it admits a set of orthonormal eigenvectors $v_1, ..., v_k$ with $X^T X v_k = \mu_k^2 v_k$ and positive eigenvalues $\mu_1^2 \geq \mu_2^2 \geq ... \geq \mu_k^2$. Now, express $\alpha$ in the eigenbasis of $X^T X$:

$$\alpha = \sum_{k=1}^{m} a_k v_k.$$

Note that since the vectors $v_i$ are orthonormal and $\alpha$ is a unit vector, we must have

$$||\langle a_1, ..., a_k \rangle|| = 1.$$

Now,

$$\langle X^T X\alpha, \alpha\rangle = \langle\sum_{k=1}^{m} a_k\, X^T X v_k, \sum_{k=1}^{m} a_k v_k\rangle = \sum_{j,k=1}^{m} a_j a_k \langle X^T X v_j, v_k\rangle \tag{1}$$

$$= \sum_{j,k=1}^{m} a_j a_k \langle \mu_k^2\, v_k, v_j\rangle = \sum_{j,k=1}^{m} a_j a_k \mu_k^2\, \langle v_k, v_j\rangle \tag{2}$$

$$= \sum_{k=1}^{n} a_k^2\, \mu_k^2. \tag{3}$$

Since $||\langle a_1, ..., a_k\rangle|| = 1$, this is maximized by setting $a_1 = 1$ and all other $a_i = 0$, yielding $\alpha = v_1$. This completes the proof □

In particular, this validates the use of right-eigenvectors of $I$ as components with high Shannon information.

We now seek additional maximally-informative components orthogonal to the first right-eigenvector of $I$. This is equivalent to constraining $a_1 = 0$ in (3); in this case, the maximum is clearly attained by setting $a_2 = 1$ and all other $x_i = 0$, resulting in $\mathbf{a} = v_2$. Continuing, we see that the right eigenvectors yield an orthonormal basis which is maximally informative in the sense outlined in the main text.

## Supplementary Note 2: Time and Memory Performance

In light of rapidly increasing single-cell dataset sizes, it is vital that emerging methods scale to large datasets. To see how SCA scales, we ran SCA for a single iteration on various-sized subsets of the T-cell from Patient 1 from Hao et al. [6], containing 9,969 T-cells and 20,739 genes. To assess scaling with increasing numbers of cells, we measure time and memory performance on slices of Patient 1's T-cell data with all genes and random cell subsets of size 1000, 2000, 3000,...,9000. Similarly, to assess scaling with increasing numbers of genes, we assess performance on slices with all cells and random gene subsets of size 4000-20000, in increments of 4000. Finally, to assess time and memory scaling with more iterations of SCA, we ran SCA for 1-5 iterations on all cells and the 5,000 most highly-variable genes. For each slice, we run three replicates to ensure robust measurements. All benchmarks were measured on an Intel Xeon Gold 6130 CPU (2.10GHz,

768 GB of RAM).

The results of this analysis, shown in Figure S1a, show that SCA's runtime and memory footprint are linear the number of cells. As described in the Methods, SCA processes data in constant-sized chunks of genes to minimize memory allocation. For these experiments, we group genes into chunks of size 1000; consequently, the memory allocation does not depend strongly on the total number of genes. SCA is necessarily slower than PCA, because it runs a singular-value decomposition analogous to PCA's on the surprisal matrix.

For further comparison, we also performed time benchmarks for CellSIUS [16], GiniClust [15], FiRE [17], and RaceID [14] on the entire Patient 1 data from Hao (9,969 T-cells and 20,739 genes; Figure S1b). We found that all of these methods have comparable runtime with the exception of RaceID, which takes nearly two hours to run.

Since each iteration of SCA runs the same code (with the exception of the first, where the initial PCA reduction must be performed), runtime scales linearly in the number of iterations (Figure S1c). Memory allocation does not increase with more iterations, since large data structures are cleared or overwritten between iterations.

## Supplementary Note 3: Performance on randomized data

A major concern with any signal-boosting approach is false positives, i.e. finding signal when none is present. For SCA, we must ensure that truly structure-less data remains so in the SCA representation.

We construct a random Gaussian dataset (i.e. each transcript is drawn at random from a standard Normal distribution) with 1,000 cells and 10,000 genes. As expected, UMAP plots derived from 20-dimensional SCA representations do not show any significant structure (Figure S3a). As a more realistic scenario, we randomly permuted each gene in the cytotoxic T-cell dataset from [27], eliminating gene-gene correlations (Figure S3b). On this data, the leading PC aligns with the highest-variance gene, in this case granulysin, so that the PCA representation loosely separates cells that express granulysin from cells that do not. SCA boosts this signal to produce a stronger separation, but does not otherwise cluster the data.

## Supplementary Note 4: Automatic determination of the multiple-testing correction factor

Transcriptional datasets often profile tens of thousands of genes, but correlations among the genes mean that the data has significantly fewer degrees of freedom. Consequently, the statistical tests performed to produce the surprisal matrix may be highly dependent. This affects the degree of multiple testing correction these scores should undergo. For example, if the genes partition into two highly-correlated modules, then each cell really only performs two tests, for the over- or under-expression of these modules. We therefore devised an empirical method to determine the appropriate multiple testing correction factor (Algorithm S3). The basic idea is to compute minimum $p$-values across all genes over many random collections of cells, from which the appropriate exponent can be determined.

More formally, we generate $N$ random neighborhoods from a dataset with $M$ genes, yielding an $N \times M$ wilcoxon $p$-values. Let $p_{ij}$ denote the $p$-value for the $j$th transcript in the $i$th random neighborhood. Now let

$$\tilde{p}_i = \min(\{p_{ij} : 1 \leq j \leq M\}).$$

Note that, by construction, for all $P \in [0, 1]$, the $p_{ij}$ values are uniform in expectation, that is:

$$\mathbb{P}(p_{ij} < P) = P.$$

If all of the genes were independent of each other, then we would expect the $\tilde{p}_i$s to have a distrution whose CDF is

$$\mathbb{P}(\tilde{p} < P) = 1 - (1 - P)^M$$

However, dependence among genes may reduce the exponent on the right hand side. To determine the correct exponent $t$, we empirically measure this CDF for our observations. Assuming without loss of generality that

$\tilde{p}_1 \leq \tilde{p}_2 \leq ... \leq \tilde{p}_N$, we have an empirical CDF satisfying:

$$\mathbb{P}_{emp}(\tilde{p} < p_i) = \frac{i}{N}.$$

Comparing this to the previous equation, we obtain a regression problem:

$$\frac{i}{N} \sim 1 - (1 - \tilde{p}_i)^t.$$

After some algebra, this becomes

$$\log(1 - \frac{i}{N}) \sim t \log(1 - \tilde{p}_i).$$

In real datasets, we indeed observe that the relationship between $\log(1 - \frac{i}{N})$ and $\log(1 - \tilde{p}_i)$ is approximately linear. Thus, a reasonable choice for $t$ is the slope of the regression line between $\log(1 - \frac{i}{N})$ and $\log(1 - \tilde{p}_i)$ for the computed $\tilde{p}_i$ (assumed to be indexed in loosely increasing order). This regression line necessarily passes through zero, so there is no need to fit an intercept. The $t$ output by Algorithm S3 is the mean-squared error estimate for this slope.

## Supplementary Note 5: Single-cell surprisal analysis

Remacle et al. [47] and Gross et al. [45] use surprisal analysis on time series micro-array data to characterize the drivers of early carcinogenesis. Their approach models the observed expression as deviation from a balance state, and uses SVD to identify transcriptional programs that describe this deviation. The authors do not generalize this approach to work on single-cell data, so it is not clear how it could be applied to reduce the dimensionality of these datasets.

To see whether this approach could work in the single-cell domain, we propose an extension: rather than considering several bulk samples from different time points, treat each individual cell as its own sample, and treat them as these authors treat time points. The "maximal energy" balance state is characterized by uniform random expression of all genes, representing no constraints. Following [45], we then define the

surprisal of a transcript as the log ratio between its observed expression in a cell and its mean expression over all cells. We then perform SVD on the resulting surprisal matrix to extract a smaller number of transcriptomic programs that define cellular behavior, yielding a lower-dimensional representation. We call this approach *sc-Surprisal*.

We applied this to the cytotoxic T-cell data from [27], producing a 20-dimensional representation (the same dimensionality as the SCA, ICA, and PCA representations produced in the main text). We find that key marker genes do not neatly separate in the resulting data; for example, CD8 T-cells mix with CD4 T-cells in the downstream UMAP plot, and F1 score analyses show that other key populations do not cleanly separate (Figure S6). We suspect that this approach does not have the statistical power to detect deviation from equilibrium in individual cells, due to the higher noise compared to bulk data. SCA boosts statistical power by considering *neighborhoods* of cells, giving context to each measurement, and allowing separation of these key populations.

We next tested whether could improve sc-Surprisal by contrasting a gene's expression with its *local* expression, rather than its expression over the entire dataset. To this end, we defined the *local surprisal* of a transcript as the log ratio between its expression and its mean expression over its 15-nearest neighbors. We then performed SVD on the resulting matrix of local surprisal values. Again, we found that this method does not separate known populations in the cytotoxic T-cell data, as assessed via UMAP plots and F1 scores (Figure S6a, b).

## Supplementary Note 6: Tests with different parameters and pre-processing protocols

Filtering to highly-variable genes is a common pre-processing step with both advantages and pitfalls. On one hand, it greatly reduces the dimensionality of the data and may remove irrelevant lowly-expressed transcripts. On the other hand, a gene that marks a rare population may have low variance, and therefore may be removed, along with any hope of finding the population. In the main text, we do not perform highly-variable gene filtering as a pre-processing step for any method except PHATE, which explicitly specifies it

in its documentation. In Figure S6a and Figure S6b, we examine the performance of each method when we filter to the top 1000 most variable genes as a pre-processing step (or, in the case of PHATE, when we fail to do so).

We find that retaining highly variable genes does not significantly improve cell type recovery by PCA, ICA, scVI, or diffusion maps. For SCA, highly-variable gene filtering causes CD4 cells to mix with CD8 cells in the downstream embedding, possibly because *CD40LG*, which correlates strongly with *CD4*, is not among the 1000 most variable genes. As expected, PHATE does not produce a meaningful embedding without highly-variable gene filtering. The best performance is attained by SCA without highly-variable gene filtering.

For linear methods like PCA, ICA, and SCA, the output dimensionality, determined by the number of leading components kept, is a key parameter. Taking too few components may collapse true clusters in the data, whereas taking too many may introduce noise. In Figure S6c, we measure F1 scores for recovery of cytotoxic T cell populations using SCA, PCA, and ICA with 1-50 components. We find that performance is relatively stable, with strongest performance around 20 components for all three linear methods.

## Supplementary Note 7: False positive rates under imputation

The potential to introduce false positives (expression of genes where none exists) is a drawback of imputation methods. To ensure that the boosts in imputation performance that SCA enables do not come at the cost of introducing false positives, we use the Splatter-generated synthetic dataset from figure 2b-e, and measured the average level of expression of marker genes for a rare population in cells from a common population after imputation. Due to Splatter's built-in noise model, there is some expression of the rare marker genes in the common population even without imputation. We found that while no method significantly increased the degree of false-positive expression, SCA-MAGIC showed an increase of expression of the rare marker genes within the rare population over MAGIC, consistent with our other imputation results (Figure S7a).

As documented in [39], some nearest-neighbor based imputation methods, including MAGIC, have the potential increase gene-gene correlations not only between marker genes of the same population, but between

non-marker genes. This can lead to false positives in downstream marker gene detection. To assess this, we measured for each imputation method the average gene-gene correlation between all pairs of marker genes, and between all pairs of non-marker genes (figure S7b). Echoing [39], we find that MAGIC increases gene-gene correlations both among marker genes and among non-marker genes. SCA-MAGIC showed more favorable results, with higher correlation among marker genes and lower correlation among non-markers than MAGIC.

We speculate that this increase in correlation among non-markers in $k$-nearest neighbor based imputation arises due to the tendency of these methods to decrease the variance of a gene's expression within a strongly-connected population, while maintaining between-group variance. This increases the dependency between gene expression and cellular population, and thus the correlation of all genes. As outlined in [39], filtering marker genes by effect size (e.g. fold-change) can mitigate this effect.

SCA itself offers an alternative representation of gene expression via its surprisal scores. These too arise from the $k$-nearest neighbor graph; however, rather than aggregating gene expression values from neighboring cells as MAGIC does, it quantifies the over-expression of each gene in the neighborhood relative to the entire dataset. Surprisal scores measure statistical significance of a gene's expression, not its estimated expression; thus, surprisal scores should not be taken as imputed gene expression values. However, their statistical nature proves an advantage in separating marker genes from non-markers: in this simluated dataset, the surprisal scores of marker genes are more correlated even than SCA-MAGIC, whereas the correlation among non-marker genes is extremely low (figure S7b). We believe that it is precisely this property that allows SCA to better identify meaningful axes of variation, since the underlying singular-value decomposition can more effectively distinguish coordinated gene programs.

**Algorithm S1:** InfoScore

**Input:** Expression matrix $X$ with $N$ cells (rows) and $M$ genes (columns); neighborhoods $A$ with $k$ neighbors per cell; multiple testing correction factor $t$

**Result:** surprisal matrix $S$

**Initialization**: Let $S[i,j] = 0$ for all $i \in 1, ..., N$ and $j \in 1, ..., M$;

**Define:** `ranksum`$(A, B)$ for vectors $A$,$B$ as the sum of the ranks of $A$ with respect to $A \cup B$

**Define:** `ranksumtest`$(A, B)$ as the two-sided $p$-value for the Wilcoxon rank-sum test of $A$ and $B$.

**for** $i \in 1, ..., N$ **do**
  **for** $j \in 1, ..., M$ **do**
    Let $A_i = \{n_1, ..., n_k\}$ denote the indices of the $k$ nearest neighbors of the $i$th cell;
    Let $G_{ij} = \{A[\ell, j] : \ell \in A_i\}$ denote the expression vector of gene $j$ for these neighbors;
    Let $\overline{G_{ij}} = \{A[\ell, j] : \ell \notin A_i\}$ denote the expression of gene $j$ for all remaining cells;
    Let $p_{ij} =$ `ranksumtest`$(G_{ij}, \overline{G_{ij}})$;
    Let $\overline{p_{ij}} = 1 - (1 - p_{ij})^t$;
    Let $S_{ij} = -\ln(\overline{p_{ij}})$;
    **if** *ranksum*$(G_{ij}, \overline{G_{ij}}) - \frac{k(k-1)}{2} < \frac{k(N-k)}{2}$ **then**
      Let $S_{ij} \leftarrow -1 * S_{ij}$;
    **end**
    **Set** $S[i, j] \leftarrow S_{ij}$
  **end**
**end**
**Return** $S$

**Algorithm S2:** SCA

**Input:** Expression matrix $X$ with $N$ cells (rows) and $M$ genes (columns); target number of components $D$; neighborhood size $k$; number of iterations $n_{it}$; multiple testing factor $t$ (optional)

**Result:** SCA reduction $X_{SCA}$ with $N$ cells (rows) and $D$ features (columns)

**if** $t$ *is not provided* **then**
  $t \leftarrow$ `NTests`$(X, k + 1)$ (empirical derivation; see Algorithm S3);
**end**
**Initialize**: $X_{SCA} =$ D-dimensional PCA reduction of $X$;
**for** $i \in 1, 2, ..., n_{it}$ **do**
  Let $A$ denote the $k + 1$-nearest neighbors graph of $X_{SCA}$ using Euclidean distance, including points as their own neighbors;
  Let $S =$ `InfoScore`$(X, A, t)$;
  Let $V$ be the $M \times D$ matrix whose columns are the first $D$ right-eigenvectors of $S$;
  Set $X_{SCA} \leftarrow XV$
**end**
**Return** $X_{SCA}$

**Algorithm S3:** NTests

**Input:** Expression matrix $X$ with $N$ cells (rows) and $M$ genes (columns); neighborhood size $k$; number of trials $T$

**Result:** Multiple-testing correction factor $t$

;

Let $A$ be a $T \times k$ matrix containing $T$ random sets of $k$ neighbor indices drawn from $1, ..., N$;

Let $S = \texttt{InfoScore}(X, A, t = 1)$;

Let $P$ be a $T \times k$ matrix defined by $P_{ij} = -\exp(|S_{ij}|)$ (matrix of $p$-values when neighborhoods are randomly chosen);

Let $Q$ be a $T$-dimensional vector of the row minima of $P$ ;

Sort $Q$ from smallest to largest;

Let $Y = \langle \frac{1}{T}, \frac{2}{T}, ..., \frac{T-1}{T}, 1 \rangle$;

Set $Y \leftarrow -\ln(1 - Y)$, element-wise;

Set $X \leftarrow -\ln(1 - Q)$, element-wise;

Let $t = \frac{\text{mean}(\{x_i y_i : i \in 1, .., T\})}{\text{mean}(\{x_i^2 : i \in 1, ...., T\})}$ denote the slope of the least-squares regression line between $X$ and $Y$.;

Set $t \leftarrow \lceil t \rceil$ (ensuring it is an integer);

**Return** $t$

## Supplementary Figures

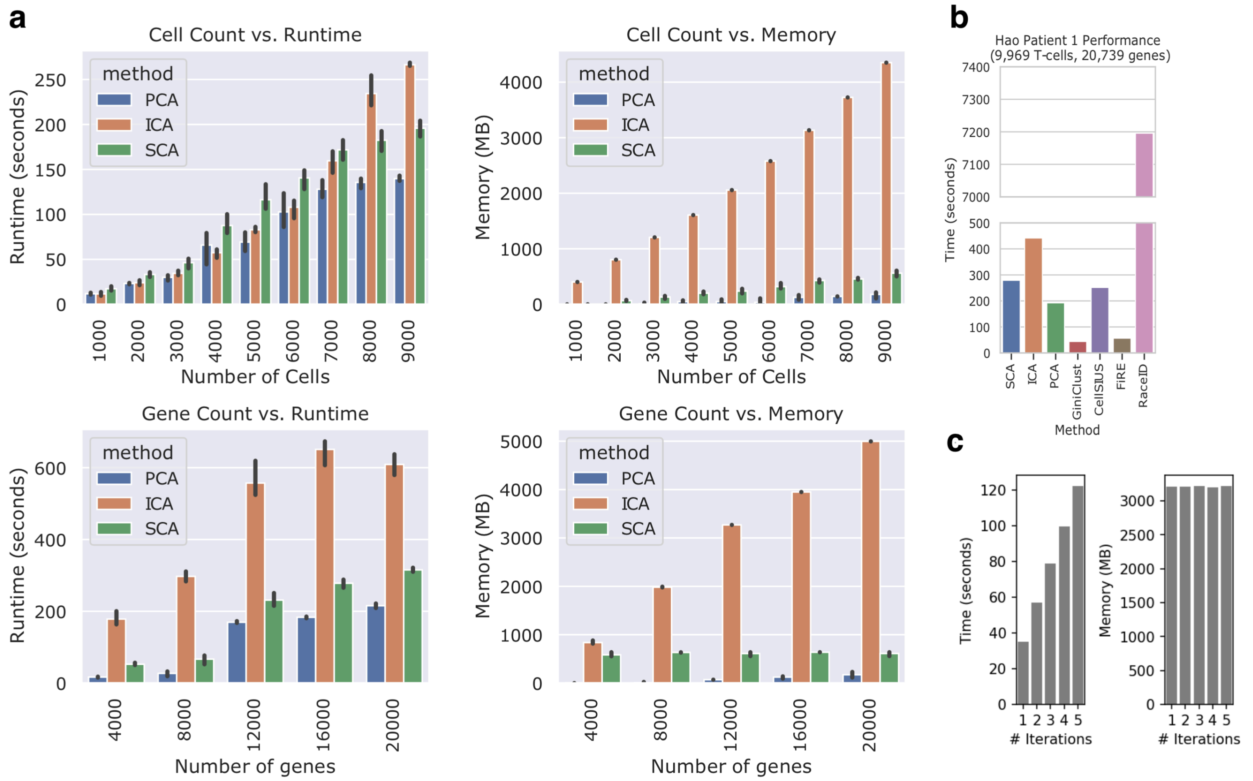


Figure S1: **a**: Time (left) and memory (right) benchmarks of SCA compared with PCA and ICA. The input dataset has 9,969 T-cells and 20,739 genes from Patient 1 in Hao [6]. In the top row, we assess scaling with increased cell numbers by taking random cell subsets of various sizes and measuring the time required to generate 50-dimensional reductions using SCA, PCA, or ICA. In the bottom row, we perform the same analysis on random gene subsets of varying sizes. Each bar represents three trials with different random subsets. **b**: Runtime comparison of SCA against existing methods for rare cell type recovery on the full patient 1 dataset. RaceID takes by far the longest, requiring approximately 2 hours to run (note the broken axis). **c**: Runtime and memory performance of 1-5 iterations SCA on Patient 1 in Hao, subset to the 5000 most highly-variable genes. Runtime scales linearly in the number of iterations, while memory allocation remains constant regardless of the number of iterations.

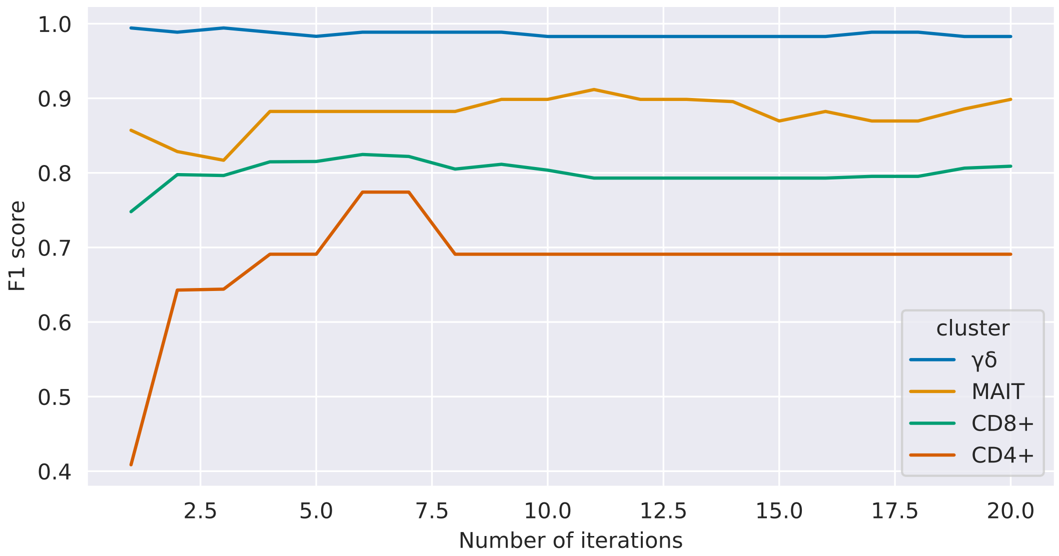


Figure S2: F1-score performance of SCA on the cytotoxic T-cell data from [27] with up to 20 iterations. Performance remains stable even after many iterations.

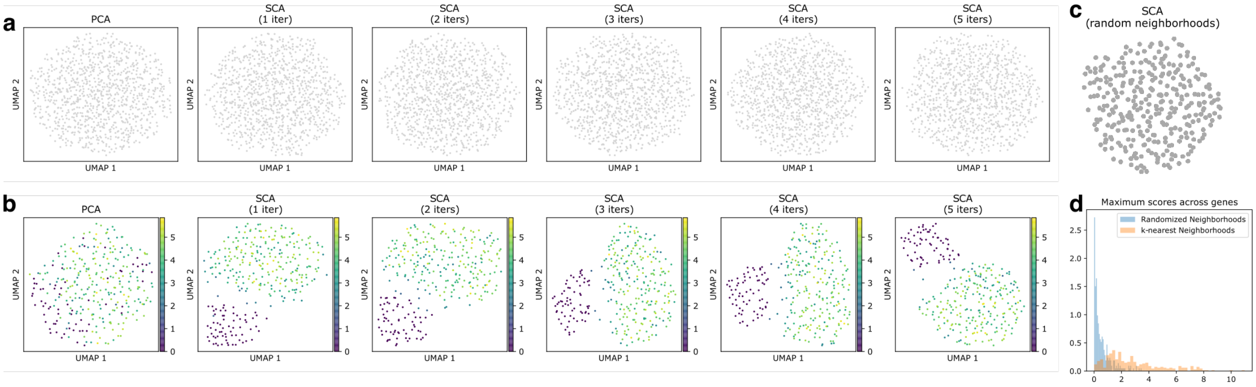


Figure S3: UMAP embeddings derived from SCA representations of "negative control" data without inter-feature relationships. We expect such data to have little structure, since most structure arises from correlations among many features. **a**: UMAP plots derived from SCA reductions of random Gaussian data with 1000 observations of 10,000 features. **b**: Reductions of scrambled cytotoxic T-cell data from [27]. Each gene was randomly permuted to eliminate gene-gene relationships. The plots are colored by granulysin expression. **c**: UMAP plot derived from 1 iteration of SCA on the cytotoxic T-cell data, but where scores are computed on random neighborhoods instead of k-nearest neighborhoods. **d**: Histogram of maximum magnitude of scores attained by each gene in any cell when neighborhoods are computer randomly (blue) or via k-nearest neighbors in PCA space (orange).

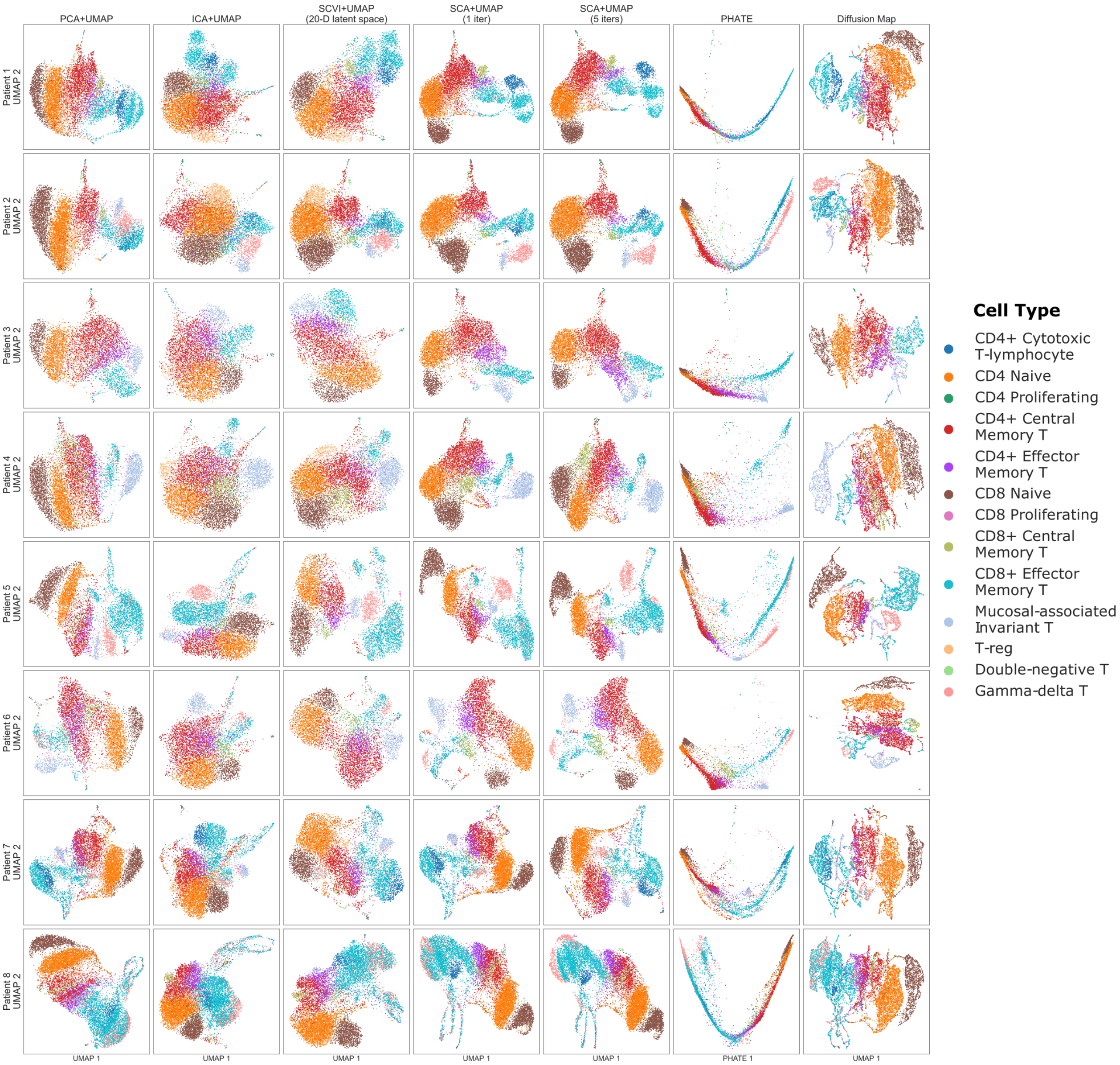


Figure S4: UMAP plots of cytotoxic T-cells for all patients in Hao et al., computed using PCA, ICA, scVI, Diffusion Maps, or SCA with 1 or 5 iterations, colored by cell type. The two-dimensional PHATE reduction is also shown (sixth column).

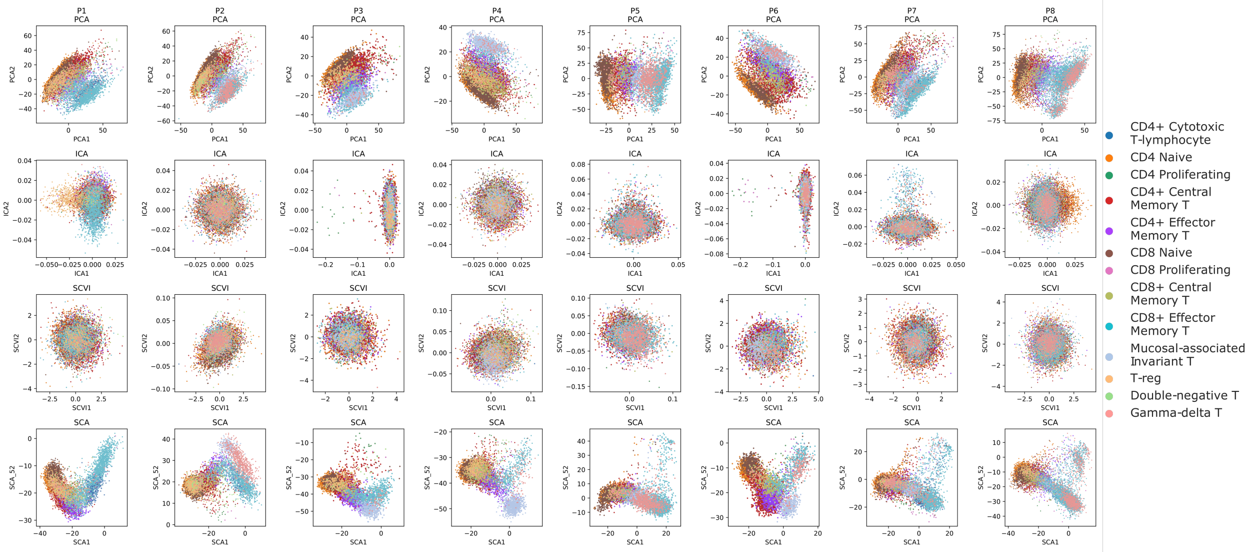


Figure S5: Component plots of cytotoxic T-cells for all patients in Hao et al., computed using PCA, ICA, scVI, or SCA with 1-5 iterations. For each reduction, the first two dimensions are plotted against each other. For SCA, we perform 5 iterations.

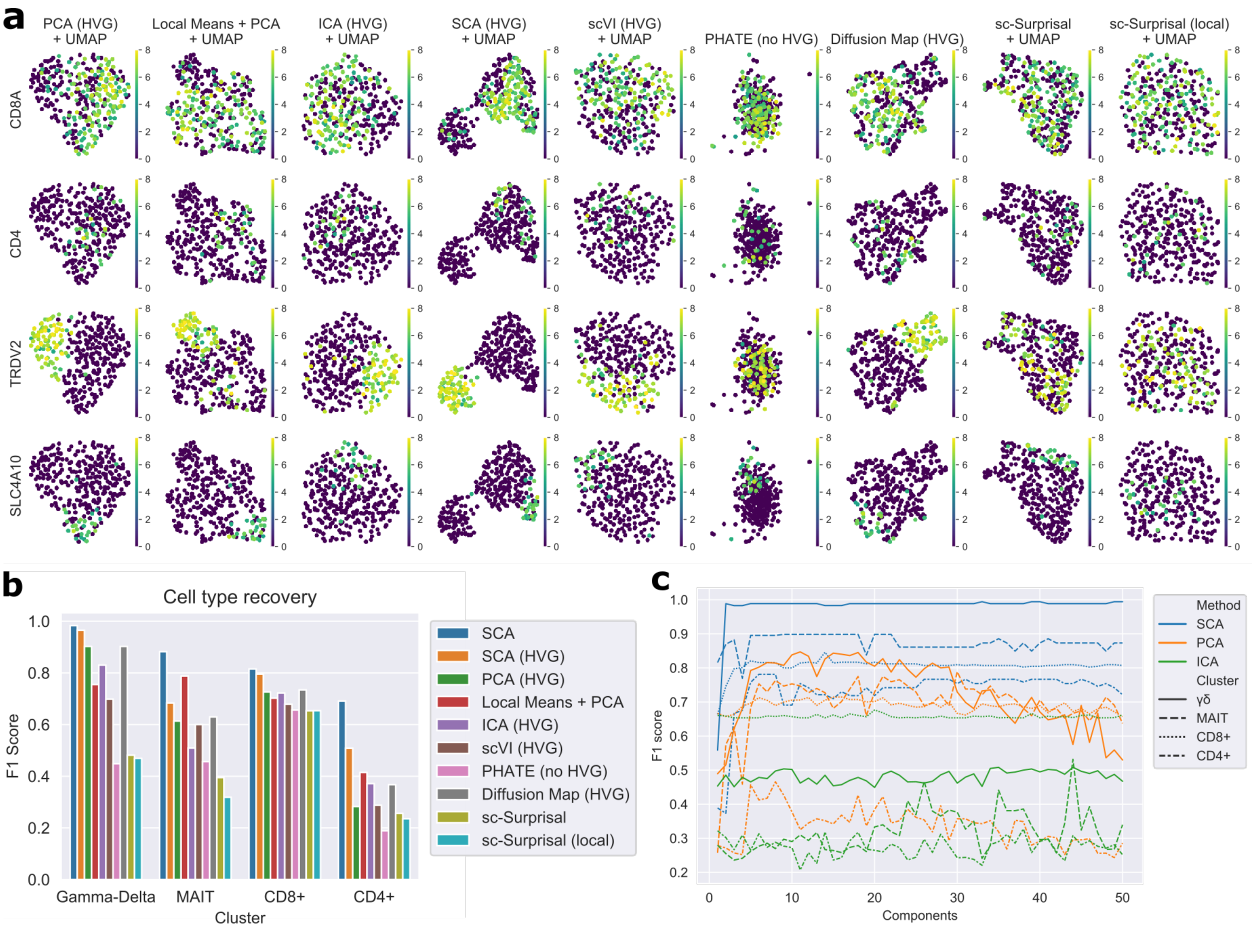


Figure S6: Performance of additional embedding strategies on the cytotoxic T-cell dataset. **a**. UMAP plots made from 20-dimensional reductions using various embedding and pre-processing steps: PCA, ICA, SCA, scVI, and a diffusion map after highly variable gene filtering; PHATE without highly variable gene filtering; PCA of mean expression values across k-nearest neighbors ("Local Means + PCA"); a single-cell analogue of surprisal analysis ([45]) where gene expressions are divided by global expression means before reduction ("sc-Surprisal"), and a version of surprisal analysis where gene expression values are divided by their mean expressions among the 15 nearest neighbors ("sc-Surprisal (local)"). **b**: F1 scores of Leiden clusterings downstream of the 20-dimensional reductions visualized in (a), against known cell types. SCA outperforms other approaches, including itself after highly variable gene selection. **c**: Effect of the number of components on recovery accuracy for known cell types using PCA, ICA, and SCA. Performance is largely stable, with SCA performing consistently better for all three types.

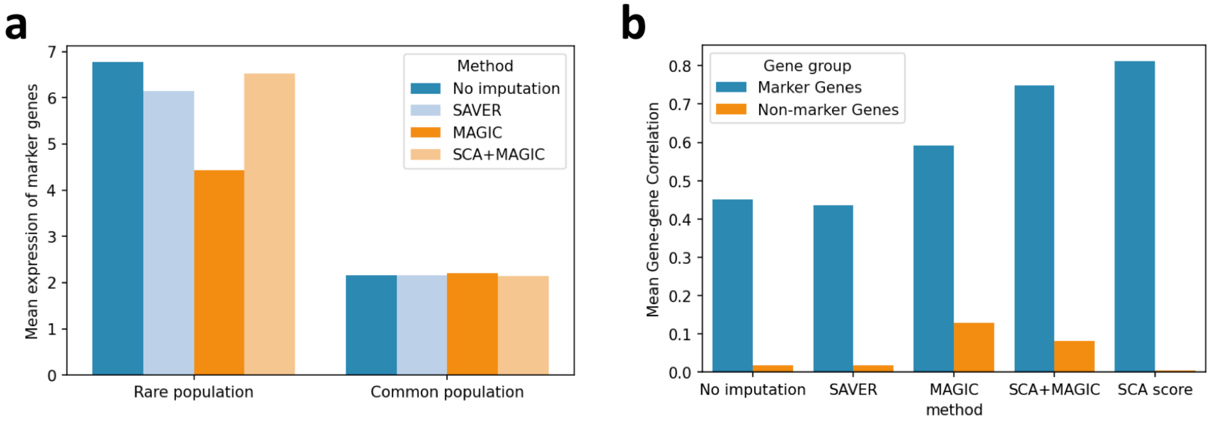


Figure S7: **a**: Mean expression of marker genes for the rare cellular population in each of the two populations from the simulated dataset described in Figure 2. Relative to MAGIC, SCA-MAGIC increases the expression of the marker genes in the rare population without increasing their expression in the common population. **b**: Average gene-gene correlation among marker genes and among non-marker genes on imputed data, and on SCA scores themselves. MAGIC and SCA-MAGIC induce a slight positive correlation among non-marker genes. However, the SCA scores themselves retain high correlation among markers without introducing correlation among non-markers.
