# Supplementary material for: SCA: recovering single-cell heterogeneity through information-based dimensionality reduction
Source: Genome Biol. 2023 Aug 25;24:195. doi: 10.1186/s13059-023-02998-7 (PMC10464206; doi:10.1186/s13059-023-02998-7)
Supplement: Supplementary file 2 — Additional file 2. Contains the review history. [file 13059_2023_2998_MOESM2_ESM.docx]

Review History

**First round of review**

**Reviewer 1**

**Were you able to assess all statistics in the manuscript, including the appropriateness of statistical tests used?**

**Yes:** The statistical tests used in this manuscript look pretty good to me.

**Were you able to directly test the methods?**

No

**Comments to author:**

DeMeo & Berger proposed a new dimensionality reduction algorithm which was designed to preserve information from rare and subtly-defined cell types better than the currently canonical ones. In general, the proposed algorithm has shown some promising results but there are multiple issues that remain to be addressed.

1. Batch effect: would the current design of SCA amplify the (inter-sample) batch effect too? Further evaluations should be performed and discussed.

2. Scalability: While I appreciate the authors' explicit discussion on the SCA's time and memory performance in Supplementary Note 2, the current reported results are based on a single iteration running which might hardly be possible in real-world scenario.

3. Hyperparameter sensitivity: During simulation, the algorithm performance suffered a considerable drop for k neighbor size >20 (Fig 2d), but no such phenomenon was observed on the real PBMC dataset (Fig 3f). Such inconsistency would raise concerns on the hyperparameter stability of SCA.

**Reviewer 2**

**Were you able to assess all statistics in the manuscript, including the appropriateness of statistical tests used?**

**Yes:** no further assessment required

**Were you able to directly test the methods?**

Yes

**Comments to author:**

Here, DeMeo and Berger present a novel method for dimensionality reduction for scRNAseq data. The approach is rooted in Shannon's information theory and this framework is used to calculate a surprise score to indicate if the presence or absence of a gene is notable given the neighbors in expression space. The authors use this surprisal score to perform a linear dimensionality reduction. Through multiple examples, they demonstrate that this approach allows them to identify small and subtly defined cell types. Given the difficulty of identifying rare cell types, this method is a welcome addition to the computational biologists' toolbox. It is based on sound mathematical principles, and as such I believe that it has the potential to be adopted by the community. Nevertheless, I believe that the following major concerns need to be addressed:

1 The authors use synthetic data to evaluate the method and this provides a ground truth to evaluate the performance of different methods. However, this only serves as a positive control, and in addition, I would like the authors to also use a negative control, i.e. a situation when no rare cell types are present, to make sure that SCA does not report false positives. Fig 2a partly addresses this question, but I would like to see an additional control to investigate this issue directly. I understand that doing the same test for a real dataset is going to be more challenging, but if the authors could come up with some clever way of doing this then it would enhance the paper since I find synthetic data to be of limited utility.

2 One aspect of PCA that is often very useful is that the eigenvalues can be used to assign importance of the corresponding components as "fraction of variance explained". I was wondering if it is possible to do something similar for SCA? From p18-19 it is clear that the SCA involves an eigenvector calculation, but as far as I can tell you are never using the eigenvalues. Is there any meaningful information in the eigenvalues? If not, it might be helpful if the authors commented on this in the manuscript.

3 Similarly, in PCA one often looks at the different features which contributes the most to each PC. I assume that something analogous could be done with SCA? If so, could the authors investigate which genes contribute the most to the top SCs for the examples in fig 3 and 4? In particular, do they correspond to the markers that you use to annotate the different cell types?

4 One of the issues with imputation methods is that they can produce false positives (e.g. Andrews and Hemberg, F1000 Research, 2019 or Hou et al, Genome Biology, 2020). I would like the authors to investigate if SCA-MAGIC also suffers from this issue.

5 As far as I can tell, this approach is not directly tied to scRNAseq data. Thus, I was wondering if it could also be applied to scATACseq and other modalities? If it does work out well, I think that the manuscript would be greatly enhanced with a small use-case. However, I can imagine that the greater number of features and higher sparsity in scRNAseq data could be problematic. In this case it would be useful if the authors could comment on this in the discussion.

Minor:

Line 331: what is "desi"?

Line 351: do you mean "complement" rather than "compliment"?

**Authors Response**

We would first like to sincerely thank all the reviewers for their insight, their positive responses to the paper, and for noting our contributions. We appreciate the constructive feedback which has certainly improved the manuscript. Main revision points are summarized below:

- We have provided new experiments on real, recently-released datasets to demonstrate that SCA does not amplify batch effects.
- We have expanded guidance on neighborhood size selection.
- We have provided further analysis of false positives in imputation results and shown that SCA does not induce them.
- We have added a brief discussion of the use of elbow plots and component loading analysis with surprisal components, and modified the code slightly to make the former more workable.
- We have added an additional subfigure to Figure S1 to demonstrate the impact of more iterations on runtime and memory footprint.
- We have made minor wording changes in several places to address reviewer concerns.

**Point-by-point responses to the reviewers’ comments:**

**Response to Reviewer 1**:

*DeMeo & Berger proposed a new dimensionality reduction algorithm which was designed to preserve information from rare and subtly-defined cell types better than the currently canonical ones. In general, the proposed algorithm has shown some promising results but there are multiple issues that remain to be addressed.*

Thank you for appreciating our results and suggesting further constructive experiments.

*1. Batch effect: would the current design of SCA amplify the (inter-sample) batch effect too? Further evaluations should be performed and discussed.*

You raise an important concern regarding batch effects and whether SCA amplifies them. We now evaluate the impact of batch on SCA’s reductions on a recently-released (inter-sample) dataset containing approximately 70,000 CD34+ immune cells from 3 donors. We find that the donor-derived batch effect was markedly *reduced* in the SCA reduction relative to the PCA reduction, and this reduction increased with more iterations of SCA. Furthermore the separation between biological cell types remained uniformly better than PCA. These analyses are newly included in a new section of the main text, and in the new **Figure 6**.

2. *Scalability: While I appreciate the authors' explicit discussion on the SCA's time and memory performance in Supplementary Note 2, the current reported results are based on a single iteration running which might hardly be possible in real-world scenario.*

Thank you for the comment. We have added a subfigure to Figure S1 showing the time and memory footprints of SCA when run with 1-5 iterations. Since the algorithm (and source code) to perform an iteration of SCA is the same regardless of whether it is the first or a subsequent iteration, run time scales linearly in the number of iterations. As large data structures (in particular the surprisal scores and neighborhood gene expressions) are cleared or overwritten between iterations, adding more iterations does not impact the memory footprint.

While these benchmarks suggest that most users will be able to run multiple iterations without difficulty, even a single iteration improves over PCA in the recovery of key cell types (for example, gamma-delta T-cells were newly recovered after just 1 iteration on the cytotoxic T-cell data). Thus, it is certainly workable to run a single iteration in a real-world scenario in the unusual event that runtime prohibits multiple iterations.

3. *Hyperparameter sensitivity: During simulation, the algorithm performance suffered a considerable drop for k neighbor size >20 (Fig 2d), but no such phenomenon was observed on the real PBMC dataset (Fig 3f). Such inconsistency would raise concerns on the hyperparameter stability of SCA.*

You note an apparent inconsistency in the hyperparameter sensitivity analyses: on our simulated dataset, SCA’s performance deteriorated when its neighborhood size exceeded 20, whereas no such drop was observed on real biological data. Indeed, the drop in performance observed in Figure 2d is entirely expected, and serves to illustrate an important consideration in neighborhood size selection: as the chosen neighborhood size approaches the size of a rare cellular population in the dataset, the algorithm loses the ability to distinguish that population, because the cells in the rare population begin to have many neighbors in different populations. Here, the rare population contains only 34 cells, so a neighborhood size greater than 20 cannot adequately capture it. This point was not fully addressed in our initial submission, and we have now expanded the accompanying section to address it.

However, we do not observe this decline in performance on the cytotoxic T-cell dataset even with neighborhood sizes as high as 50. This too is unsurprising, for two reasons. First, most of the cellular populations in this dataset have more than 50 cells, so larger neighborhood sizes are workable. Second, we suspect that unlike in the synthetic dataset, there are other biological patterns besides direct marker genes which serve to separate the various populations from each other, leading to a stronger starting signal for SCA to amplify. In fact, our synthetic dataset is intentionally more difficult than most real datasets. We designed it this way in order to push SCA to its limits, which we believe is of great service to potential users.

As both of our robustness analyses show excellent separation of cell types with neighborhood sizes as small as 5, and stable performance at least up to half the size of the smallest cellular population, we believe that neighborhood size need not be a concern for the vast majority of users. We have nevertheless expanded both accompanying sections to explicitly outline these guidelines.

**Response to Reviewer 2**:

*Reviewer #2: Here, DeMeo and Berger present a novel method for dimensionality reduction for scRNAseq data. The approach is rooted in Shannon's information theory and this framework is used to calculate a surprise score to indicate if the presence or absence of a gene is notable given the neighbors in expression space. The authors use this surprisal score to perform a linear dimensionality reduction. Through multiple examples, they demonstrate that this approach allows them to identify small and subtly defined cell types. Given the difficulty of identifying rare cell types, this method is a welcome addition to the computational biologists' toolbox. It is based on sound mathematical principles, and as such I believe that it has the potential to be adopted by the community. Nevertheless, I believe that the following major concerns need to be addressed:*

Thank you very much for appreciating SCA’s contribution.

*1 The authors use synthetic data to evaluate the method and this provides a ground truth to evaluate the performance of different methods. However, this only serves as a positive control, and in addition, I would like the authors to also use a negative control, i.e. a situation when no rare cell types are present, to make sure that SCA does not report false positives. Fig 2a partly addresses this question, but I would like to see an additional control to investigate this issue directly. I understand that doing the same test for a real dataset is going to be more challenging, but if the authors could come up with some clever way of doing this then it would enhance the paper since I find synthetic data to be of limited utility.*

You requested a benchmark of SCA when no rare cells are present to ensure that SCA does not produce false clusters. This is the intent of Supplementary Note 3 and the accompanying Figure S3, which assess the behavior of SCA on data without intrinsic structure (either from random Gaussian noise, or from scrambling the expression values of each gene in a real dataset). In both cases, SCA does not turn up unexpected clusters, although both PCA and SCA mildly separate granulysin-expressing from non granulysin-expressing cells in the scrambled cytotoxic T-cell data (since this gene has high expression and variance relative to other genes, it produces a separation even after scrambling, which PCA and SCA detect). We realize that these results were not sufficiently emphasized in the main text, and have now given them more emphasis.

*2 One aspect of PCA that is often very useful is that the eigenvalues can be used to assign importance of the corresponding components as "fraction of variance explained". I was wondering if it is possible to do something similar for SCA? From p18-19 it is clear that the SCA involves an eigenvector calculation, but as far as I can tell you are never using the eigenvalues. Is there any meaningful information in the eigenvalues? If not, it might be helpful if the authors commented on this in the manuscript.*

Reviewer #2 notes that PCA’s linear nature enables useful features like elbow plots (plotting the eigenvalues of the input matrix in order to determine the appropriate number of components). Although our initial submission did not mention it, it’s perfectly reasonable to do this with SCA by plotting the eigenvalues of the surprisal matrix. We have now explicitly stated this (*Overview of results*), and have made a minor modification to SCA’s source code to provide users easy access to the eigenvalues for this purpose.

*3 Similarly, in PCA one often looks at the different features which contributes the most to each PC. I assume that something analogous could be done with SCA? If so, could the authors investigate which genes contribute the most to the top SCs for the examples in fig 3 and 4? In particular, do they correspond to the markers that you use to annotate the different cell types?*

As you note, it is sometimes useful to examine the top-loaded genes in each principal component. The same can be said of surprisal components, and SCA’s interface optionally gives users access to these loadings for this purpose. We did not mention this in our initial submission and have now done so in *Overview of Results*.

*4 One of the issues with imputation methods is that they can produce false positives (e.g. Andrews and Hemberg, F1000 Research, 2019 or Hou et al, Genome Biology, 2020). I would like the authors to investigate if SCA-MAGIC also suffers from this issue.*

False positives are indeed a concern of imputation methods. As requested, we have performed an additional benchmark to ensure that the improved imputation performance of SCA-MAGIC does not come at the cost of increased false positives. A new analysis of synthetic data with a known ground truth (Supplementary Note 7 and the new Figure S7) shows that SCA does not increase the incidence of false positives over other methods.

*5 As far as I can tell, this approach is not directly tied to scRNAseq data. Thus, I was wondering if it could also be applied to scATACseq and other modalities? If it does work out well, I think that the manuscript would be greatly enhanced with a small use-case. However, I can imagine that the greater number of features and higher sparsity in scRNAseq data could be problematic. In this case it would be useful if the authors could comment on this in the discussion.*

As you note, nothing about SCA makes it applicable only to scRNA-seq data, and with the increasing availability of multi-modal single-cell data, there is potential for other applications. While application to other modalities is certainly possible, we have not tested SCA nearly as extensively in these modalities, and so refrain from explicitly recommending its use there. Most likely, SCA would work best on these modalities with modality-specific choices. For example, in the recently-developed single-cell ATAC-seq, latent semantic indexing takes the place of SVD as a reduction engine. This substitution is readily accomplished via a custom reducer in SCA’s [object-oriented API](https://shannonca.readthedocs.io/en/latest/objapi.html), but further benchmarking would be required.

*Minor:*

*Line 331: what is "desi"?*

Designed–fixed.*Line 351: do you mean "complement" rather than "compliment"?*
Yes, thanks for the catch. Fixed in two places!

**Response to the reviewer who had difficulty running SCA:**

We very much appreciate your feedback. It is surprising to us that you had difficulty running SCA, as we have deployed it on several different machines and Python installations without difficulty, and have tried to make it easy for others to do so, for example via the [Quick start](https://shannonca.readthedocs.io/en/latest/quickstart.html#with-scanpy) page in the online documentation. Though we did not receive specific details on the issue you encountered, we can offer the following general troubleshooting guidelines:

- Pull from the github repository to ensure that your version of SCA is up to date. Although we have made no major changes since submission, this will ensure we are running the same code.
- Ensure that the Python version is at least 3.0, and that the installations of scanpy, numpy, scipy, and sklearn are up to date.
- When running the reduce function, ensure that the input is either a CSR sparse matrix (scipy.sparse.csr_matrix) or a dense numpy array, with one row per cell and one column per gene. Coercion to sparse matrices is easy via scipy.sparse.tocsr()
- When running reduce_scanpy, ensure that the input is a scanpy anndata object.
- Ensure that the data type of the input is either an integer or float.
- Double-check that the code follows the docstring for the relevant function: [reduce](https://shannonca.readthedocs.io/en/latest/generated/shannonca.dimred.reduce.html#shannonca.dimred.reduce) or [reduce scanpy](https://shannonca.readthedocs.io/en/latest/generated/shannonca.dimred.reduce_scanpy.html#shannonca.dimred.reduce_scanpy).

We would be able to give more tailored help if we knew more details of the issue you encountered. Please feel free to submit an issue to our [github](https://github.com/bendemeo/shannonca/issues) (using an anonymous new account, if desired), and we will respond promptly.

**Second round of review**

**Reviewer 1**

I'd thanks the authors for their work to address my concerns well. I believe it's now ready to go.

**Reviewer 2**

The authors have addressed all but one of the issues that I raised in a satisfactory manner. What I am not yet happy about is regarding imputation. The authors only looked at marker genes showing up in the common population. However, the issue that I am more concerned about is the imputation resulting in the creation of new markers. That is, some genes that were previously not markers end up having their expression profile changed to such an extent that they end up being reported as markers. I would like for the authors to investigate this phenomenon as well in their supplementary note.

**Authors Response**

**Point-by-point responses to the reviewers’ comments:**

**Response to** **Reviewer 2:**

*The authors have addressed all but one of the issues that I raised in a satisfactory manner. What I am not yet happy about is regarding imputation. The authors only looked at marker genes showing up in the common population. However, the issue that I am more concerned about is the imputation resulting in the creation of new markers. That is, some genes that were previously not markers end up having their expression profile changed to such an extent that they end up being reported as markers. I would like for the authors to investigate this phenomenon as well in their supplementary note.*

We thank this reviewer for their feedback, which has again improved the manuscript. Their point about artificial marker genes induced by imputation was salient, and the two sources listed in their original review offered many illuminating examples as well as guidance on how to detect these artificial markers.

We have now expanded Supplementary Note 7 and Figure S7 to better address this issue. Following Andrews and Hemberg (doi:10.12688/f1000research.16613.2), we measured gene-gene correlation on a dataset with two populations generated using Splatter, and compared the correlation of imputed values among marker genes to that among non-markers. We found that, compared to MAGIC, SCA+MAGIC produced a higher correlation among marker genes while reducing the correlation among non-marker genes (see the new Figure S7b).

However, consistent with Andrews and Hemberg’s observations, both k-NN smoothing methods produced a higher correlation among non-markers than SAVER. Upon reflection, we believe this is a consequence of MAGIC’s neighborhood-smoothing approach, which by nature reduces the variance among groups of nearby cells (discussed in Supplementary Note 7). Consequently, even a small difference in gene expression between two regions or connected components of the k-nearest neighbor network can create an apparent differential expression signal, since between-region variance is preserved while within-region variance is reduced. To mitigate this concern, one could impose a fold-change cutoff when calling differentially-expressed genes, or perhaps add a small term to the computed within-group variances when using methods like the *t*-test to assess significance of differential expression.

This reflection led to another potentially useful insight, which we have also added to Supplementary Note 7. In the process of dimensionality reduction, SCA generates a *surprisal matrix* with the same shape as the input, quantifying the degree to which each gene is overexpressed in each cell’s neighborhood. This matrix should not be viewed as an imputed version of the data, since its entries do not represent counts. However, by design, surprisal scores zero out genes with uniform expression over the dataset while accentuating those that vary. Consequently, in our Splatter-generated data, we find very high correlation in surprisal scores among marker genes, and virtually none at all among non-markers (Figure S7b). This should provide further reassurance to the reviewer’s concerns. Notably, this property underpins SCA’s strong performance in separating rare or subtly-defined cell types.

**Third round of review**

**Reviewer 2**

The authors have addressed my remaining concern and I am happy to recommend for publication.
